# Reversible dehydrogenation and rehydrogenation of cyclohexane and methylcyclohexane by single-site platinum catalyst

**DOI:** 10.1038/s41467-022-28607-y

**Published:** 2022-03-01

**Authors:** Luning Chen, Pragya Verma, Kaipeng Hou, Zhiyuan Qi, Shuchen Zhang, Yi-Sheng Liu, Jinghua Guo, Vitalie Stavila, Mark D. Allendorf, Lansun Zheng, Miquel Salmeron, David Prendergast, Gabor A. Somorjai, Ji Su

**Affiliations:** 1 The Molecular Foundry, Lawrence Berkeley National Laboratory, Berkeley, CA 94720 USA; 2 State Key Laboratory of Physical Chemistry of Solid Surfaces, Collaborative Innovation Center of Chemistry for Energy Materials, and Department of Chemistry, College of Chemistry and Chemical Engineering, Xiamen University, 361005 Xiamen, China; 3 Chemical Sciences Division, Lawrence Berkeley National Laboratory, Berkeley, CA 94720 USA; 4 Department of Chemistry, University of California-Berkeley, Berkeley, CA 94720 USA; 5 Advanced Light Source, Lawrence Berkeley National Laboratory, Berkeley, CA 94720 USA; 6 Sandia National Laboratories, Livermore, CA 94551 USA; 7 Materials Science and Engineering Department, University of California-Berkeley, Berkeley, CA 94720 USA

**Keywords:** Heterogeneous catalysis, Catalytic mechanisms, Porous materials

## Abstract

Developing highly efficient and reversible hydrogenation-dehydrogenation catalysts shows great promise for hydrogen storage technologies with highly desirable economic and ecological benefits. Herein, we show that reaction sites consisting of single Pt atoms and neighboring oxygen vacancies (V_O_) can be prepared on CeO_2_ (Pt_1_/CeO_2_) with unique catalytic properties for the reversible dehydrogenation and rehydrogenation of large molecules such as cyclohexane and methylcyclohexane. Specifically, we find that the dehydrogenation rate of cyclohexane and methylcyclohexane on such sites can reach values above 32,000 mol_H2_ mol_Pt_^−1^ h^−1^, which is 309 times higher than that of conventional supported Pt nanoparticles. Combining of DRIFTS, AP-XPS, EXAFS, and DFT calculations, we show that the Pt_1_/CeO_2_ catalyst exhibits a super-synergistic effect between the catalytic Pt atom and its support, involving redox coupling between Pt and Ce ions, enabling adsorption, activation and reaction of large molecules with sufficient versatility to drive abstraction/addition of hydrogen without requiring multiple reaction sites.

## Abstract (web summary)

Developing highly efficient and reversible hydrogenation-dehydrogenation catalysts shows great promise for hydrogen storage technologies. Here the authors develop a highly efficient and reversible de/rehydrogenation single-site platinum catalyst which exhibits great promise for hydrogen storage technologies with cyclic alkanes/aromatics as liquid organic hydrogen carriers.

## Introduction

Metal particles on various supports play a central role in energy conversion reactions and chemical transformations by heterogeneous catalysis. Ideally, the support immobilizes the particles reducing their mobility to prevent sintering, and favoring chemical stabilization^1^. Active supports, consisting of reducible oxides participate in the catalytic reaction through metal-support interactions involving charge transfer between metal particles and support, harboring multi-functional active centers, and mediating spillover of reactants and products^2–4^. Recently, the creation of single-site catalysts^5–7^, where single metal atoms are anchored on support by bonding to ligands such as N or O, has opened up a new research frontier in the catalysis field^8–10^. The isolated metal sites provide a bridge between heterogeneous and homogeneous catalysis^11^. Reduction of the size of metal nanoparticles down to a single atom achieves the maximum efficiency of utilization of expensive noble metals, with gains in activity, stability, and selectivity^12–14^. However, the lack of an ensemble of other active sites adjacent to the single noble metal atom may prevent surface reactions that involve large molecules^15,16^. Recent studies demonstrated that “ensemble effects” between the single metal atom and neighboring oxygen vacancies can favor oxygen transfer and adsorption of reactants, enabling catalysis reactions of the functional groups on large molecules with higher efficiency^17–19^. The beneficial effect of oxygen vacancy sites next to the noble metal^12,14^ motivated us to further explore its possibilities with the goal to discover new reactions and to accelerate the development of next-generation single-site catalysts.

Hydrogen is an ideal fuel for clean energy, replacing traditional hydrocarbon feedstocks. It is also used in polymer electrolyte membrane fuel cells (PEMFCs) because of its high mass-energy density of 141.6 MJ/kg, three times higher than that of gasoline, while producing water as the only by-product^20–22^. The challenges of these applications are associated with the difficulties of efficient hydrogen storage, which today are mostly based on compression and liquefaction technologies, introducing safety concerns and low storage density, as well as transportation, boil-off losses, and other high-cost problems^23^. Liquid organic hydrogen carriers (LOHCs) represent an attractive alternative to store hydrogen in chemical bonds and can address a number of limitations of present technologies^24^. Reversible LOHC systems are always composed of pairs of hydrogen-poor and hydrogen-rich organic compounds that store hydrogen by hydrogenation and dehydrogenation^25^, which has a high potential for mobile applications^26^. Cycloalkanes and aromatics with one or more six-membered rings are promising reversible LOHC systems. These stable compounds have high storage capacities (between 6 and 7 wt.% H_2_) and can be dehydrogenated/rehydrogenated under relatively mild conditions^27,28^. Supported Pt nanoparticle catalysts are industrially mature and superior heterogeneous catalysts used in hydrocarbon refineries^29,30^. Based on a sulfated Pt nanoparticle catalyst (S-Pt/Al_2_O_3_), Chiyoda Corporation developed a LOHC system with dehydrogenation/rehydrogenation of methylcyclohexane (MCH)/toluene and demonstrated its feasibility and advantage for hydrogen storage and transportation^31^. Nevertheless, the high cost and low natural abundance of Pt limited its further application. Downsizing of Pt from nanoparticle to single atoms is an effective method to maximize the Pt atom efficiency and reduce this component of the capital investment.

In this work, we show the successful fabrication and demonstration of isolated single Pt atoms on CeO_2_ (Pt_1_/CeO_2_) that is highly efficient and reversible for dehydrogenation/rehydrogenation of large cyclic hydrocarbons. As we will show, compared with traditional Pt nanoparticle catalysts (several nm in size on CeO_2_), the single Pt atom catalyst displays significantly higher catalytic performance for both dehydrogenation and rehydrogenation reactions. In particular, the turnover frequency (TOF) of cyclohexane and methylcyclohexane on such single Pt sites could reach >32,000 mol H_2_ per mol Pt per hour (5.5 g_H2_ g_Pt_^−1^ min^−1^), which is 309 times that of commercial Pt/Al_2_O_3_ (5 wt.%) catalyst. Key to this high performance is the discovery that oxygen vacancies next to the Pt atom site facilitate adsorption of these large cyclic molecules and stepwise dehydrogenation reactions driven by a super-synergistic effect involving redox coupling between Pt and Ce atoms.

## Results and discussion

### Preparation and characterization of isolated single Pt sites on CeO_2_ (Pt_1_/CeO_2_)

The Pt_1_/CeO_2_ catalyst was fabricated by a modified ascorbic acid (AA)-assisted reduction method, described in detail in the methods section^32^. Porous CeO_2_ nanorods were synthesized and employed as supports to isolate Pt single sites due to their high specific area. Inductively Coupled Plasma Optical Emission Spectroscopy (ICP-OES) measurements revealed that the final content of Pt was 0.15 wt.%.

**Fig. 1:**
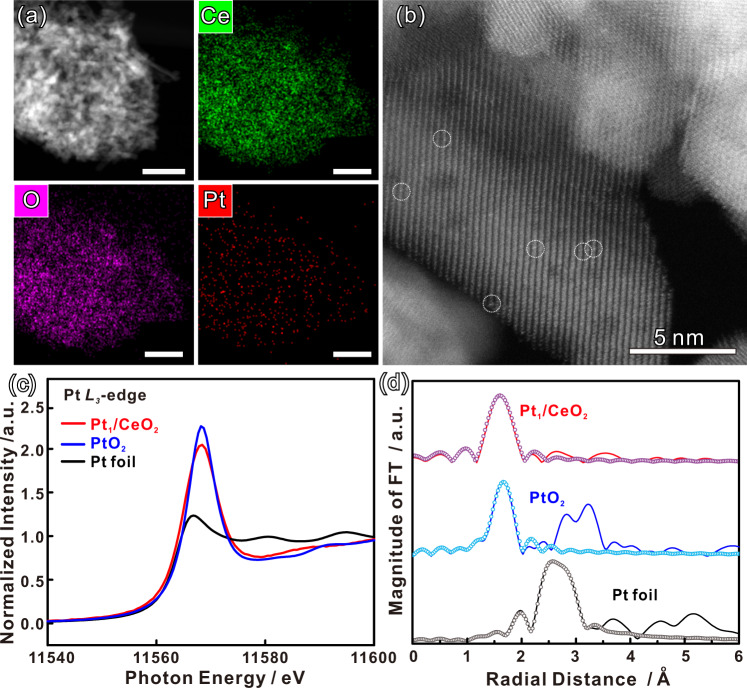
Morphology and structural characterization of Pt_1_/CeO_2_ catalysts. **a** High-angle annular dark-field scanning transmission electron microscopy (HAADF-STEM) images and corresponding elemental map images of the Pt_1_/CeO_2_ catalyst (scale bars = 50 nm). **b** Cs-corrected HAADF-STEM images of the Pt_1_/CeO_2_ catalyst. The brighter dots (circled) are from Pt single sites. **c** Normalized Pt *L*_*3*_-edge XANES spectra, and (**d**) *k*^*3*^-weighted Fourier transform EXAFS spectra (*L*_*3*_-edge) of Pt in Pt_1_/CeO_2_, PtO_2_, and bulk Pt foil at the Pt *L*_*3*_-edge. The circles are fit to the data.

After deposition of Pt on CeO_2_, no peaks from Pt were observed in the powder X-ray diffraction (XRD) pattern, due to the low Pt content and small size of the particles that may be present (Supplementary Fig. 1). Moreover, no difference was observed, relative to the original CeO_2_ nanorods, in bright-field transmission electron microscopy (TEM) images, indicating that the number of such Pt nanoparticles or clusters is below the detection limit (Supplementary Fig. 1). However, elemental mapping of Pt_1_/CeO_2_ catalyst by HAADF-STEM, shows that Pt is present and uniformly distributed on the CeO_2_ nanorods (Fig. 1a). More importantly, Cs-corrected HAADF-STEM images reveal the presence of isolated Pt single sites (white circles), which are brighter compared to surrounding Ce due to their larger atomic number, and dispersed throughout the porous CeO_2_ support, as shown in Fig. 1b^5,32^. Important additional information was obtained from X-ray absorption spectroscopy at the Pt *L*_3_ edge, in experiments with Pt foils, PtO_2_ as a reference, and with the Pt_1_/CeO_2_ catalyst. The near-edge fine structure (XANES) provides insights into the valence state of Pt (Fig. 1c), while the extended X-ray adsorption fine structure part (EXAFS) provides information on the local bonding geometry^33^. The EXAFS data from the Pt_1_/CeO_2_ catalyst show only one peak from Pt-O bonds around 1.5 Å, but no Pt-Pt bonds could be detected, as shown by comparison with the reference bulk PtO_2_ and Pt foil (Fig. 1d). Details of the EXAFS fitting results are listed in Supplementary Table 1. The combined evidence from Cs-corrected HAADF-STEM and EXAFS strongly support the conclusion that the Pt in the Pt_1_/CeO_2_ catalyst is largely present as isolated single atoms on CeO_2_ while no metallic particles or clusters are present in detectable amounts. In more detail, compared with the PtO_2_ reference, although there is not an obvious white line shift of Pt_1_/CeO_2_ due to the low Pt concentration on the surface and detector limitation, the lower energy shoulder (Fig. 1) indicates that the Pt oxidation state in Pt_1_/CeO_2_ is lower than +4^34^, in agreement with the ambient-pressure X-ray photoelectron spectroscopy (AP-XPS) results. These low oxidation state Pt species are critical to our proposed mechanism below, especially in the initial stage of the reaction.

### Dehydrogenation of cyclohexane

**Fig. 2:**
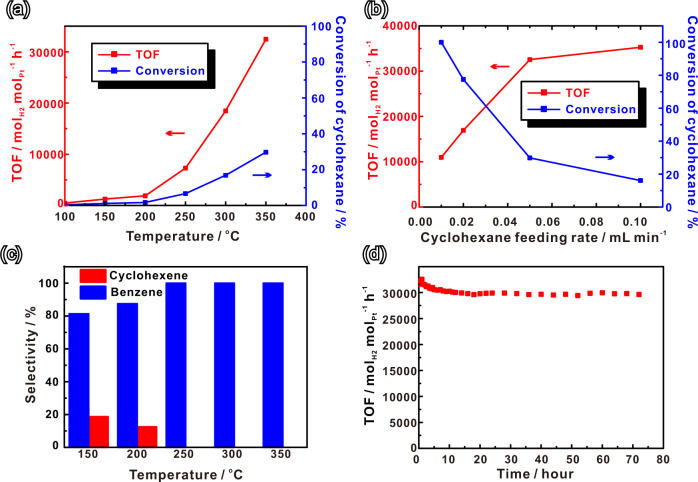
Catalytic cyclohexane dehydrogenation performance of Pt_1_/CeO_2_. **a** Turnover frequency (TOF) of hydrogen production per mol Pt of Pt_1_/CeO_2_ and conversion of cyclohexane at different temperatures. Reaction conditions: (**b**) turnover frequency (TOF) of hydrogen production per mol Pt of Pt_1_/CeO_2_ and conversion of cyclohexane at 350 °C for different cyclohexane feeding rates. **c** Selectivity of cyclohexane dehydrogenation at different temperatures. **d** Turnover frequency (TOF) of hydrogen production per mol Pt of Pt_1_/CeO_2_ at 350 °C during 72 h of reaction.

**Fig. 3:**
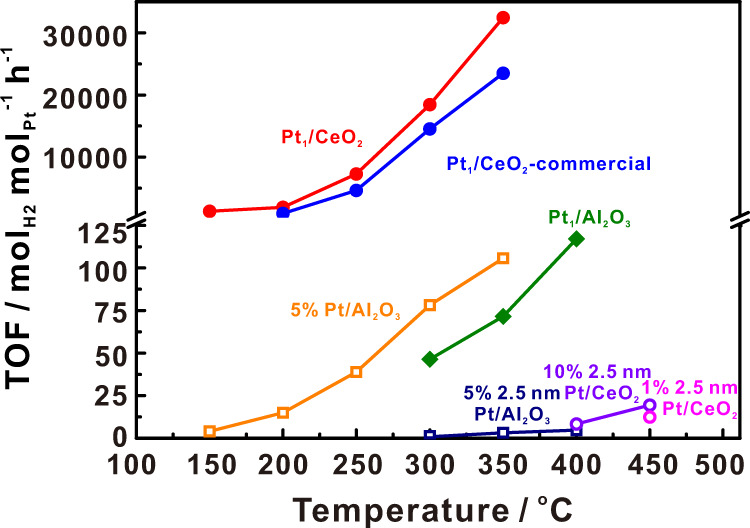
Comparison Pt_1_/CeO_2_ catalytic performance with other catalysts. Turnover frequency (TOF) of hydrogen production per Pt site of different catalysts as a function of temperature. The circle symbols correspond to supports with cyclohexane adsorption ability while square symbols correspond to supports with no cyclohexane adsorption ability. Solid symbols denote single Pt site catalysts while hollow symbols denote Pt nanoparticle catalysts.

As a reversible LOHC system, cyclohexane/benzene is ideal for the storage and transportation of hydrogen. Here we used the as-prepared Pt_1_/CeO_2_ single-site catalyst for the catalytic cyclohexane dehydrogenation reaction (Supplementary Fig. 2). The catalyst showed excellent activity compared to that of Pt nanoparticles on CeO_2_ (2.5 nm Pt/CeO_2_ and 7.0 nm Pt/CeO_2_), which were synthesized according to previous work (Supplementary Fig. 3)^26^. At temperatures below 100 °C all the catalysts were inactive. When the temperature increased to 100 °C hydrogen was generated at measurable rates from the dehydrogenation of cyclohexane on the Pt_1_/CeO_2_ catalyst. With further increase in temperature, the TOF of hydrogen generation by cyclohexane dehydrogenation increased, reaching 32,477 mol_H2_ mol_Pt_^−1^ h^−1^ (5.5 g_H2_ g_Pt_^−1^ min^−1^) at 350 °C (Fig. 2a), which is 309-fold higher than that of commercial Pt particles on aluminum oxide catalysts with 5% loading (Fig. 3). In contrast, no catalytic activity was detected with Pt nanoparticles of 2.5 nm and 7.0 nm size on CeO_2_ until the temperature increased to 400 °C (Fig. 3, Supplementary Table 2). To further assess the catalytic performance of Pt_1_/CeO_2_, we measured the TOF of hydrogen generation and conversion from cyclohexane at 350 °C at different cyclohexane feed rates (Fig. 2b). At low feed rates (0.01 mL/min), the conversion of cyclohexane reached 100%, with a TOF around 10,900 mol_H2_ mol_Pt_^−1^ h^−1^ (1.9 g_H2_ g_Pt_^−1^ min^−1^). Alternatively, a higher TOF around 35,400 mol_H2_ mol_Pt_^−1^ h^−1^ (6.0 g_H2_ g_Pt_^−1^ min^−1^) was achieved at feed rates of 0.1 mL/min. As shown in the literature, the equilibrium constant (*K*_eq_) of the dehydrogenation of cycloalkanes under an ambient pressure only depends on the reaction temperature^35^. And the equilibrium conversion for the dehydrogenation of cyclohexane and methylcyclohexane could reach 99% above 593 K. In our study, the highest conversion at 350 °C is around 30% (Fig. 2a), which is much lower than the equilibrium conversion. Therefore, the reaction TOF could reach values as high as 32,000–35,200 mol H_2_ per mol Pt per hour. These results illustrate the excellent catalytic activity of Pt_1_/CeO_2_ catalyst, and its potential for process scale-up. Moreover, the single-site Pt_1_/CeO_2_ catalyst exhibited remarkably high selectivity towards benzene production in the cyclohexane dehydrogenation reaction. At temperatures of 150 °C and 200 °C, the major dehydrogenated product (more than 80%) was benzene with <20% of cyclohexene detected. When the temperature was above 250 °C, essentially only dehydrogenation product is benzene (Fig. 2c).

For practical applications, stability and recyclability are important parameters when evaluating long-term catalyst performance. After 72 h at 350 °C with 3 mL/h cyclohexane feeding rate, the Pt_1_/CeO_2_ catalyst still maintains more than 90% of its original activity (Fig. 2d), the small decrease of activity results from the loss of some Pt species on CeO_2_^26^. The recyclability of Pt_1_/CeO_2_ catalyst was assessed by cyclic cyclohexane dehydrogenation experiments (Supplementary Fig. 4). The results indicate that there is no obvious activity decay during four cycles of cyclohexane dehydrogenation under different temperatures. EXAFS was also employed to confirm that the structure of Pt_1_/CeO_2_ remained stable after long reaction times and after repeated cycles of catalytic reaction. No apparent difference was found between used and freshly prepared Pt_1_/CeO_2_ catalysts, indicating that during reaction the fine structure of the Pt_1_/CeO_2_ remains unchanged (Supplementary Fig. 5). We attribute the high stability to the embedded nature of the Pt into the CeO_2_ surface layer, which is produced by depositing it on a high surface area of CeO_2_ at relatively low Pt loading amounts^36,37^. In addition, the abundant Ce^3+^ species further increase the stability of Pt_1_/CeO_2_ catalysts^38^. However, increasing the temperature to 400 °C resulted in a decrease in reaction activity as a result of some Pt aggregation and carbon deposition, as revealed by EXAFS (Supplementary Figs. 5, 6).

### Cyclohexane dehydrogenation on different catalysts

It has been reported that single-site catalysts have limitations for reactions involving large molecules due to the lack of surrounding ensemble sites to accommodate the molecule and its products. However, our results show that the single-site Pt_1_/CeO_2_ is 309 times more active than Pt particles supported on CeO_2_ or Al_2_O_3_, which ranks the highest among the reported catalysts (Supplementary Fig. 7)^39^. To understand this effect and the reaction mechanism, a comparison of the performance of Pt and its different ensemble size effects in different supports is needed. We tested the dehydrogenation reaction of cyclohexane on a series of catalysts such as different Pt nanoparticles of different sizes on CeO_2_, single-site Pt on Al_2_O_3_, and commercial Pt catalysts (5% Pt/Al_2_O_3_) (Fig. 3, Supplementary Fig. 8, and Supplementary Table 2). We found that on different Pt nanoparticles sizes (2.5 nm and 7 nm) loaded on CeO_2_, different pretreatments, and different Pt content, there was no hydrogen detected until the temperature was higher than 400 °C. Moreover, for single-site Pt on Al_2_O_3_ (Pt_1_/Al_2_O_3_), which was synthesized following literature reports^40^, we found that although the Pt is also in single-atom form, its catalytic activity is much lower than that of Pt_1_/CeO_2_, indicating the aforementioned super-synergistic role played by the support in case of Pt_1_/CeO_2_.

To further elucidate the role of the support in the dehydrogenation reaction we used in situ DRIFTS to study cyclohexane adsorption and desorption^41^. The results showed that the as-prepared Pt_1_/CeO_2_ support exhibited much stronger adsorption of cyclohexane as compared to that of Pt_1_/Al_2_O_3_ (Supplementary Fig. 9). Due to the strong interaction between CeO_2_ and cyclohexane, after stopping the flow of cyclohexane and purging with pure N_2_ for 40 min, there are still two peaks around 2930 cm^−1^ and 2852 cm^−1^ which are attributed to adsorbed cyclohexane, while for the Al_2_O_3_ support, these peaks disappeared after 15 min of N_2_ purging. Also, commercial CeO_2_ powders (Supplementary Fig. 10) were studied for cyclohexane adsorption using FTIR. We found that cyclohexane adsorption is very weak and all the cyclohexane desorbed during 10 min pure N_2_ purging. To confirm the cyclohexane adsorption site on CeO_2_, XPS was used to determine the Ce oxidation state in commercial CeO_2_ powders compared to that in the as-prepared CeO_2_ (Supplementary Fig. 11)^42^. The Ce 3*d* XPS spectra are very sensitive to its oxidation state, showing 10 peaks which are attributed to Ce^3+^ (881.0, 883.4, 899.3 and 903.1 eV) and Ce^4+^ (882.2, 888.4, 898.1, 900.7, 907.3 and 916.7 eV), which were used to calculate the concentration of Ce^3+^ in CeO_2_^32,43^ The results show that the O vacancies (V_O_) in our CeO_2_ nanorods is higher than that in the commercial powders. To further verify that V_O_ promote cyclohexane adsorption, we loaded Pt on commercial CeO_2_ by the same ascorbic acid (AA) assisted reduction method, which created some V_O_ sites on CeO_2_ at the same time (Supplementary Table 2). The Pt *L*_*3*_-edge EXAFS showed that similar to the Pt_1_/CeO_2_ catalyst, Pt on the commercial CeO_2_ particles was also a single site (Supplementary Fig. 5). The in situ DRIFTS (Supplementary Fig. 9) showed that the higher Vo concentration improved the cyclohexane adsorption ability, causing the Pt_1_/CeO_2_-commerical catalyst activity to be comparable to the activity of Pt_1_/CeO_2_ catalyst at lower temperature (200 °C).

### Ambient-pressure X-ray photoelectron spectroscopy (AP-XPS)

**Fig. 4:**
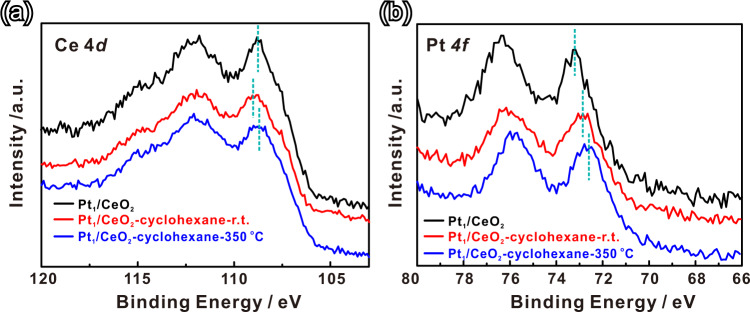
In situ AP-XPS of Pt_1_/CeO_2_ catalyst. **a** Ce 4*d* and (**b**) Pt 4*f* APXPS spectra of Pt_1_/CeO_2_ catalysts in different conditions_._ The black, red, and blue lines are Pt_1_/CeO_2_ in vacuum, in 0.1 Torr cyclohexane at room temperature and 0.1 Torr cyclohexane at reaction temperature of 350 °C, respectively.

To identify the nature of the active site in Pt_1_/CeO_2_ during reaction, AP-XPS was employed to measure the valence state change of Pt and Ce during cyclohexane dehydrogenation. Fig 4a shows the Ce 4*d* spectra of Pt_1_/CeO_2_ catalysts under different conditions^44^. We expect the lowest binding energy peak for Ce^4+^ at 109.3 eV and for Ce^3+^ the stronger peak in this range appears at 108.0 eV (with a much weaker peak at 105.4 eV)^45^. Based on the measured line shape, we can see a slightly increasing Ce^3+^ population with cyclohexane dosing and increased temperature, which was also confirmed by the NEXAFS results at the Ce *M*_*4,5*_ edge after absorbing cyclohexane (Supplementary Fig. 12). Knowing from our FTIR results (Supplementary Fig. 9) that cyclohexane adsorption is driven by oxygen vacancies in our catalyst, after dosing 0.1 Torr of cyclohexane vapor, the binding energy of Ce^4+^ 4*d* shifted to slightly higher energy, which we ascribe to surface polarization of CeO_2_ due to adsorbates (Fig. 4a). With further increase of the temperature to 350 °C, which causes hydrogen generation from cyclohexane dehydrogenation, and driving off adsorbates, we notice that the Ce^4+^ 4*d* peak shifts back to a lower energy^46^. The Pt 4 *f* XPS reveals lower binding energy peaks ranging from 73.2 to 72.9 to 72.6 eV, slightly above measured PtO peaks at 72.2 eV (PtO_2_ appears at 74.5 eV)^47^. One possibility is that we may be seeing Pt^3+^ (reported 4 *f* binding energy of Pt^3+^ is 73.3 eV)^48^ due to chemical bonds formed with Pt^2+^ active sites (possibly remaining ligands on the as-prepared sample)—see our mechanism below. By contrast with Pt *L*_3_ XANES, lower binding energy 4 *f* XPS is extremely surface sensitive and highlights a lower oxidation state dominance at the surface. After introducing 0.1 Torr cyclohexane, and then increasing the temperature, we see a slight reduction in Pt 4 *f* binding energy, which is evident as a slight reduction in valence charge due to the presence of adsorbates in our density functional theory (DFT) calculations. We saw no evidence for metallic Pt in any of these spectra (peak at 70.8 eV) which speaks to the stability of our catalyst at high operating temperatures.

### Mechanism

The incorporation of the Pt atom into the ceria surface, by replacing a surface Ce atom, moves well beyond the strong metal-support interactions that might be expected for supported metal nanoparticles or clusters. Therefore, the traditional metal-support interaction and reaction models which were built based on the surface/interface of metal nanoparticles and support, can no longer precisely describe the interactions and reaction occurring in the local environment of a single metal atom^49,50^. Our results, combining the characterization data reported above and DFT calculations of model catalyst reactions, show that the combination of (1) the incorporated Pt site, (2) a neighboring oxygen vacancy, and (3) the variable redox of Ce provide the necessary flexibility to catalyze multiple reactions. Specifically, the combination of these three components in the single-site Pt_1_/CeO_2_ catalyst enables: adsorption, activation, and reaction of reactant (cyclohexane); H species spillover; and H_2_ reformation. We refer to this as a super-synergistic effect, by contrast with synergistic catalysis^51^, in which two distinct catalysts can enhance reactions between two different reactants.

The in situ FTIR and AP-XPS results indicate that the ceria support, especially the abundance of oxygen vacancies, plays a significant role during cyclohexane adsorption and dehydrogenation. In a perfect crystal of CeO_2_, the formal oxidation state of Ce is Ce^4+^ with no oxygen vacancies and Pt substitution for Ce would nominally place it in the same Pt^4+^ oxidation state, but, in combination with an oxygen vacancy^52,53^, our DFT calculations show that the Pt *5d* unoccupied orbitals lie just lower in energy than the Ce *4f* orbitals, and so the two excess valence electrons released by the O vacancy reduce Pt^4+^ to Pt^2+^, which adopts the signature of the planar PtO_4_ coordination of Pt^2+^ by breaking bonds with two ceria O atoms (Supplementary Fig. 20). We propose that the super-synergistic coupling between the single-atom Pt and the ceria support with oxygen vacancy is driven by redox coupling (or disproportionation) between both the metal ions:

$${{{\rm{Pt}}}}^{2+}+{{{{\rm{Ce}}}} ^{4+}}\to{{{{\rm{Pt}}}}^{3+}}+{{{{\rm{Ce}}}}^{3+}},$$

which is vital for forming chemical bonds between formally radical intermediates and the single-site Pt. We propose two dominant reactions for cyclohexane dehydrogenation:

(1) Hydrogen abstraction and Pt–C bond (denoted as Pt–C bond) formation:

$$({{{{{\rm{C}}}}}}_{6}{{{{{\rm{H}}}}}}_{2n})\ast +{{{{{\rm{Pt}}}}}}^{2+}+{{{{{\rm{O}}}}}}^{2-}+{{{{{\rm{2Ce}}}}}}^{4+}{\to}\,{{{{{\rm{Pt}}}}}}^{3+}-({{{{{\rm{C}}}}}}_{6}{{{{{\rm{H}}}}}}_{2n-1})+{{{{{\rm{OH}}}}}}^{-}+{{{{{\rm{2Ce}}}}}}^{3+},$$

where *n* = 6, 5, 4. Removal of a neutral H atom from the organic group would nominally produce two radicals. The (neutral) H atom sheds its electron to form Ce^3+^ and joins the oxide dianion to produce a hydroxide ion (OH^−^) (Supplementary Figs. 15, 16). The *5d*^*8*^ electronic configuration of Pt^2+^ cannot bind with the organic radical, so it sheds an electron to form another Ce^3+^ and accepts the radical’s electron into a now half-filled 5*d* orbital, forming a strong bond with the C atom of the adsorbate.

(2) H abstraction and Pt < C = C bond (denoted as Pt < C=C bond) formation:

$${{{{{{\rm{Pt}}}}}}}^{3+} 	-\!({{{{{{\rm{C}}}}}}}_{6}{{{{{{\rm{H}}}}}}}_{{{2n}}-1})+{{{{{{\rm{Ce}}}}}}}^{3+}+{{{{{{\rm{O}}}}}}}^{2-}+{{{{{{\rm{2Ce}}}}}}^{{4+}}\to{{{{{\rm{Pt}}}}}}}^{2+}{ < \,{{{{{\rm{C}}}}}}}_{6}{{{{{{\rm{H}}}}}}}_{{{2n}}-2}\\ 	 +{{{{{{\rm{Ce}}}}}}}^{4+}+{{{{{{\rm{OH}}}}}}}^{-}+{{{{{{\rm{2Ce}}}}}}}^{3+},$$

where *n* = 6, 5, 4 again. In this case, the Pt reverts to its original Pt^2+^ oxidation state and bonds with the $\pi$ electrons of a C=C double bond, effectively bonding with both the carbon atoms. In the process, the Pt atoms rotates its bonding plane to meet the C=C double bond by breaking two O bonds and forming one new O bond (Supplementary Fig. 17). The high cation coordination of the cubic ceria crystal provides this flexibility for the Pt to adjust its bonding environment and make these two very different bonds to accommodate saturated and unsaturated hydrocarbons both present in dehydrogenation products.

We also propose an analogous super-synergy exists for the dehydrogenation of the catalyst surface, to dissociate H_2_ or recombine protons and electrons into H_2_ molecules via a Pt–H bound intermediate (Supplementary Fig. 18):

$$({{{{{\rm{H}}}}}}_{2}){\ast} +{{{{{\rm{Pt}}}}}}^{2+}+{{{{{\rm{2Ce}}}}}}^{4+}\to{{{{{\rm{Pt}}}}}}^{3+}-{{{{{\rm{H}}}}}}+{{{{{\rm{OH}}}}}}^{-}+{{{{{\rm{2Ce}}}}}}^{3+},$$

$${{{{{\rm{2OH}}}}}}^{-}+{{{{{\rm{Pt}}}}}}^{2+}+{{{{{\rm{2Ce}}}}}}^{3+}\to{{{{{\rm{Pt}}}}}}^{3+}-{{{{{\rm{H}}}}}}+{{{{{\rm{OH}}}}}}^{-}+{{{{{\rm{O}}}}}}^{2-}+{{{{{\rm{2Ce}}}}}}^{3+}.$$

To summarize this analysis derived from DFT calculations, the super-synergy results from: the incorporation of a Pt atom by substitution at a surface Ce site; a neighboring oxygen vacancy; and the variable redox of Ce. The major features of super-synergy are as follows: (1) The exchange of electrons between the active Pt site and the ceria support permits facile switching of the function of the Pt atom in binding directly to a single C atom of a radical adsorbate versus binding to a pair of atoms in a C=C double bond of a non-radical adsorbate; (2) The disproportionation between Pt and Ce oxidation states facilitates H abstraction or addition via spillover (with similar duality for dissociation or recombination of H_2_; and (3) the reducible ceria support acts as a reservoir for abstracted hydrogen to permit serial dehydrogenation of adsorbed aromatics followed by reformation and release of H_2_.

**Fig. 5:**
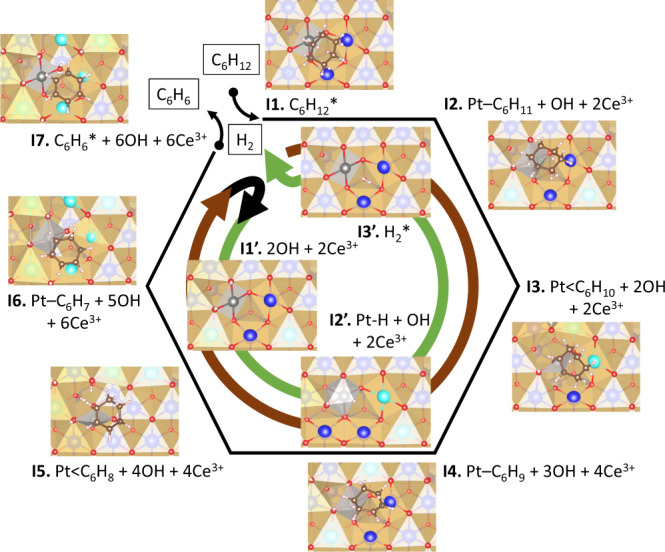
Cyclohexane dehydrogenation pathway on Pt_1_/CeO_2_ catalyst. Proposed cyclohexane dehydrogenation pathway (brown arrow) on Pt_1_/CeO_2_ at an O vacancy (V_O_) involving H abstraction to the catalyst surface (as OH^−^) with associated formation of small polarons (Ce^3+^) followed by recombination and release of H_2_ (green arrow). Reaction intermediates are labeled as **I1**-**I7** (dehydrogenation) and **I1’**-**I3’** (H_2_ release), with relative energies provided in Supplementary Fig. 19 of the Supporting Information. Structural snapshots are provided for each intermediate. Ce^4+^: light blue (sub-surface) and dark blue (surface); Ce^3+^: turquoise; Pt: gray; O: red; H: white.

Now, we can sequentially combine these reactions (Fig. 5) to reveal how Pt_1_/CeO_2_ favorably dehydrogenates cyclohexane to produce benzene, without the need for multiple catalytic sites due to the super-synergy just described. Both cyclohexane and benzene exhibit reasonable physisorption energies (−18.8 and −15.9 kcal/mol, respectively, from DFT calculations) due to the presence of oxygen vacancies and validated above using DRIFTS and X-ray spectroscopy. This is welcome, as our previous study showed that single metal sites had poor adsorption of molecules such as CO^26^. However, the mechanism here is clearly dominated by the successive exothermic formation of strongly bound surface hydroxy anions (Supplementary Fig. 19). In principle, in the absence of kinetic data (i.e., activation energies), the thermodynamic driving force is present to advance the dehydrogenation. Usually, H_2_ reformation from a metal oxide surface is the rate-determining step for hydrocarbon dehydrogenation and requires high reaction temperatures^54,55^, fortunately, due to the presence of the single-atom Pt site, the energy cost for H_2_ reformation and desorption is reduced significantly (Supplementary Fig. 19). At functional temperatures, the saturated local environment around each Pt single-site can be purged by releasing H_2_ gas and the next cyclohexane molecule can be processed. Similarly, for hydrogenation of benzene, the favorable dissociation of physisorbed H_2_ will enrich the vicinity of the Pt single-site with available hydrogen that can then begin to resaturate benzene. Now it is the uphill process of cyclohexane reformation that requires high temperatures.

As shown in Supplementary Fig. 9, the commercial CeO_2_, which has a low oxygen vacancy concentration, has very poor capacity for cyclohexane adsorption. With the ascorbic acid treatment, Pt single sites are formed together with O vacancies in the support, which promotes the adsorption of cyclohexane. The calculated adsorption energies of cyclohexane on CeO_2_ slab, CeO_2_ slab with one oxygen vacancy, Pt_1_/CeO_2_ slab and Pt_1_/CeO_2_ slab with one oxygen vacancy are −10.5, −14.8, −11.8, −18.8 kcal/mol, respectively. However, the super-synergy between Pt and Ce cations is also vital. This explains why on the inert support of Al_2_O_3_, there is no cyclohexane adsorption (no vacancies), and dehydrogenation of cyclohexane at low temperature does not occur (Al^3+^ cannot easily change oxidation state). In contrast, the Pt nanoparticles on Al_2_O_3_ show detectable dehydrogenation reaction of cyclohexane, due to typical metal surface catalysis^48^. These results clearly outline stark differences between the dehydrogenation mechanisms of single Pt sites and Pt nanoparticles. At last, compared with cyclohexane, intermediates such as cyclohexene and cyclohexadiene have been reported to be more active and easier to dehydrogenate to benzene^52,53^. Indeed, our calculated energetics (Supplementary Fig. 19) indicate that these intermediate cyclic alkenes (**I3** and **I5** in Fig. 5) are increasingly stable on the path towards generating the triene, benzene. The only uphill process is the generation of the penultimate intermediate, C_6_H_7_. However, given that all species are strongly bound to the surface, this helps explain the observed high selectivity (100%) towards benzene, at high temperature (Fig. 2b).

### Benzene hydrogenation

**Fig. 6:**
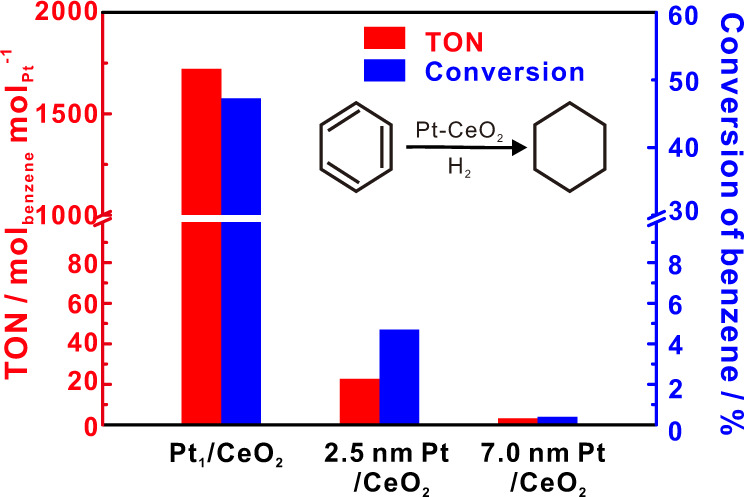
Catalytic benzene hydrogenation performance of on Pt_1_/CeO_2_ and related catalyst. Catalytic benzene hydrogenation on Pt-CeO_2_ catalysts (Pt_1_/CeO_2_, 2.5 nm Pt/CeO_2_, and 7 nm Pt/CeO_2_). Turnover number (TON) of benzene conversion per Pt site for 8 h and conversion of benzene on different Pt-CeO_2_ catalysts. Reaction conditions: 40 mg catalysts dispersed in 3 mL *n*-heptane; addition of 100 μL of benzene in 27.22 atm of hydrogen gas at 120 °C and stirred for 8 h.

An ideal LOHC system would feature both the hydrogen-rich and hydrogen-deficient organic compounds as liquids. And the usage of a proper catalytic system would allow for the efficient release of hydrogen by promoting the dehydrogenation reaction, and recovery of the hydrogen depleted by dehydrogenation. Hence, hydrogenation of benzene by the same single Pt_1_/CeO_2_ catalyst was investigated. As shown in the literature, high temperature and low pressure favor the dehydrogenation, and, reversibly, high H_2_ pressure and relatively low temperature favor the rehydrogenation. Therefore, in our study, which is different from these dehydrogenation reactions, dehydrogenation was carried out in gas-phase fixed-bed reactor with ambient pressure at a temperature of 350 °C, the hydrogenation reactions were carried out in a liquid-phase batch reactor with a low reaction temperature (60–120 °C) and high H_2_ pressure (3.40–27.22 atm). Interestingly, for the benzene hydrogenation reaction, the single-site Pt_1_/CeO_2_ catalyst showed excellent catalytic activity, much higher than that of Pt nanoparticle catalysts (2.5 nm Pt/CeO_2_ and 7 nm Pt/CeO_2_) (Fig. 6)^56^. We further tested Pt_1_/CeO_2_ catalytic properties under different reaction conditions (Supplementary Fig. 13). Under 27.22 atm H_2_ and at 120 °C, with a molar ratio of H_2_ to benzene of 5 to 1 in the batch reactor, we found that 50% of the benzene could be rehydrogenated. Our experiments indicate that conversion of benzene increases with reaction temperature and hydrogen pressure, as well as with the ratio of H_2_ to benzene. Therefore, a higher conversion (>99%) of rehydrogenation of benzene or toluene could be anticipated by further optimizing the reaction conditions (especially with high H_2_ pressure).

### Reversible dehydrogenation/hydrogenation of methylcyclohexane/toluene

**Fig. 7:**
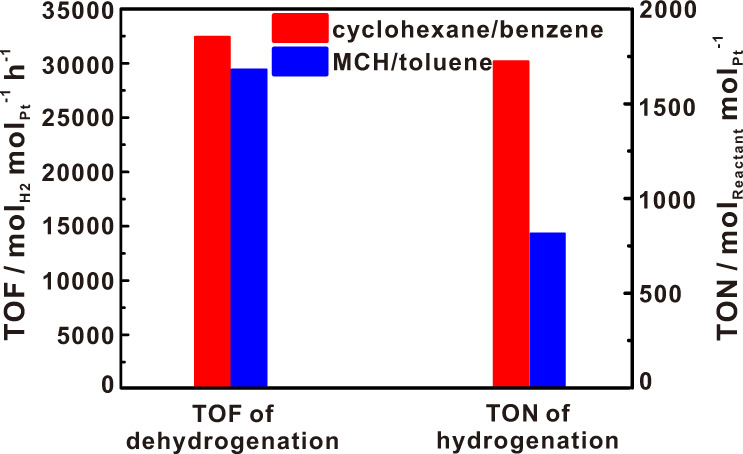
Comparisons of dehydrogenation and hydrogenation efficiency of different molecules. Dehydrogenation reaction conditions: 100 mg catalyst mixed with 500 mg sand; Gas flow: N_2_ 30 ml/min; Methylcyclohexane feeding rate: 3 mL/h; temperature: 350 °C. Hydrogenation reaction conditions: 40 mg catalyst dispersed in 3 mL *n*-heptane; added 100 μL toluene under 27.22 atm hydrogen at 120 °C and stirred for 12 h.

In the practical application of liquid cyclohexane as carrier to store and transport hydrogen, the dehydrogenation product, benzene, is classified as a carcinogen. By comparison, toluene, the dehydrogenation product of methylcyclohexane, although with a similar structure as benzene, has much lower toxicity. Strikingly, we found that, during the methylcyclohexane dehydrogenation, the Pt_1_/CeO_2_ single-site catalyst also exhibited outstanding catalytic activity for dehydrogenation. Under the reaction conditions (350 °C), the TOF of hydrogen production was about 29,300 mol_H2_ mol_Pt_^−1^ h^−1^ without any other by-products. As for toluene hydrogenation, 800 mol_toluene_ mol_Pt_^−1^ was achieved under relatively mild reaction conditions (Fig. 7 and Supplementary Fig. 14). That means this efficient single-site Pt_1_/CeO_2_ catalyst can be also applied in methylcyclohexane/toluene reversible hydrogen storage and transportation ^57,58^.

In summary, we demonstrated the advantages of ensemble reaction sites formed by a single Pt atom and surrounding oxygen vacancies, Pt_1_/CeO_2_, for the dehydrogenation and rehydrogenation of large molecule reactants such as cyclohexane and methylcyclohexane. Compared to nanoparticles catalysts (2.5 nm and 7.0 nm size Pt particles on CeO_2_ and commercial 5% Pt/Al_2_O_3_), the Pt_1_/CeO_2_ catalyst exhibits greatly enhanced reaction rates of cyclohexane dehydrogenation, 309 times higher than that of conventional supported Pt nanoparticles. At the same time, the single-site Pt_1_/CeO_2_ catalyst also has significantly higher activity in the reaction of benzene hydrogenation. Moreover, this single-site catalyst was highly efficient in the dehydrogenation and hydrogenation of other aromatics, such as methylcyclohexane/toluene system for hydrogen generation and storage.

The combined results of in situ DRIFTS, APXPS, and EXAFS presented here demonstrate that the electronic properties and catalytic activity of our single-site Pt_1_/CeO_2_ catalyst can be precisely assigned to specific interactions between Pt atoms and neighboring surface atoms of the substrate. The mechanism predicted by our DFT studies is described as super synergistic. Unlike Pt nanoparticle catalysts, our single-site Pt_1_/CeO_2_ catalyst facilitates strong adsorption of large reactants due to surface oxygen vacancies that provide direct access to binding to the Pt site. Redox coupling between Pt and Ce ions and switchable O coordination environments permit alternating binding to radical and unsaturated by-products of H abstraction/addition. Spillover of H atoms to the CeO_2_ (111) surface is reversible via the Pt site and the same redox switching mechanism. And so, this combination can facilitate multiple reactions at a single site. Our results expand the application range of single-site catalyst to include large molecular reactants. They also highlight the versatility of a single-site catalyst for driving multiple reactions, paving the way for further rational design of highly efficient catalysts for sustainable energy generation and storage.

## Methods

### Catalyst preparation

The isolated single Pt sites on CeO_2_ (Pt_1_/CeO_2_) were fabricated by a modified ascorbic acid (AA)-assisted reduction method. The porous CeO_2_ nanorods were first synthesized according to previous reports. 500 mg as-prepared CeO_2_ was dispersed in 175 mL distilled water, 1 mmol (0.176 g) AA was added, and the solution was stirred at room temperature for 3 h. The collected products were washed with distilled water several times and dried in vacuum, denoted as CeO_2_-AA. Second, 140 mg CeO_2_-AA powder was dispersed in 55 mL distilled water and 0.4 mL (10 mg/mL) H_2_PtCl_6_ solution was added to it. After stirring for 3 h at room temperature, the products were collected and washed with water. After drying in vacuum, the products were calcined at 300 °C for 1 h in air to remove excess AA.

### Dehydrogenation of cyclohexane

The catalytic activity of catalysts toward hydrogen production from cyclohexane was performed in a continuous flow reactor. In a typical catalytic measurement, the 100 mg catalyst was placed in a U-shape fixed-bed flow reactor after mixing with 500 mg white quartz (50–70 mesh particle size). The feed of 30 mL/min N_2_ regulated by a mass flow controller to be at 1 atm and feed of 3 mL/h cyclohexane controlled by an injection pump were mixed together and directed towards the catalysts. The temperature of bed was monitored by a K-type thermocouple controlled by a PID 679 controller. The products were analyzed online by HP 5890 GC (hayesep D column and hayesep Q column) equipped with TCD and FID detector. Besides, the liquid products were also collected and measured by NMR (AV-700).

The turnover frequency (TOF) is calculated using the following equation:

1 $${{{{{\rm{TOF}}}}}}=\frac{{{{{{\rm{Hydrogen}}}}}}\,{{{{{\rm{Production}}}}}}}{{{{{{\rm{Number}}}}}}\,{{{{{\rm{of}}}}}}\,{{{{{\rm{active}}}}}}\,{{{{{\rm{Pt}}}}}}\,{{{{{\rm{Sites}}}}}}\times {{{{{\rm{time}}}}}}}$$

### Hydrogenation of benzene

The catalytic activity of benzene hydrogenation was performed in BioTage Endeavor parallel pressure reactor. In a typical catalytic measurement, 40 mg catalyst was dispersed in 3 mL *n*-heptane, then 100 μL benzene was added to the solution. After stirring the reaction mixture at 120 °C for 8 h under 27.22 atm hydrogen, the product was analyzed by NMR (AV-700).

The conversion and turnover number (TON) are calculated using the following equations:

2 $${{{{{\rm{Conversion}}}}}}=\frac{{{{{{\rm{Output}}}}}}\,{{{{{\rm{of}}}}}}\,{{{{{\rm{Cyclohexane}}}}}}}{{{{{{\rm{Input}}}}}}\,{{{{{\rm{of}}}}}}\,{{{{{\rm{Benzene}}}}}}}\times 100 \%$$

3 $${{{{{\rm{TON}}}}}}=\frac{{{{{{\rm{Cyclohexane}}}}}}\,{{{{{\rm{Production}}}}}}}{{{{{{\rm{Number}}}}}}\,{{{{{\rm{of}}}}}}\,{{{{{\rm{active}}}}}}\,{{{{{\rm{Pt}}}}}}\,{{{{{\rm{Sites}}}}}}\,\times \,{{{{{\rm{time}}}}}}}$$

## Supplementary information

Supplementary Information

Peer Review File
