# Supplementary material for: Reversible dehydrogenation and rehydrogenation of cyclohexane and methylcyclohexane by single-site platinum catalyst
Source: Nat Commun. 2022 Mar 1;13:1092. doi: 10.1038/s41467-022-28607-y (PMC8888751; doi:10.1038/s41467-022-28607-y)
Supplement: Supplementary file 1 — Supplementary Information [file 41467_2022_28607_MOESM1_ESM.pdf]

Supplementary Information

Reversible Dehydrogenation and Rehydrogenation of Cyclohexane and Methylcyclohexane by Single-Site Platinum Catalyst

Luning Chen,[1,2,3,†] Pragya Verma,[1,†] Kaipeng Hou,[4,†] Zhiyuan Qi,[3] Shuchen Zhang,[3] Yi-Sheng Liu,[5] Jinghua Guo,[5] Vitalie Stavila,[6] Mark D. Allendorf,[6] Lansun Zheng,[2] Miquel Salmeron,[3,7] David Prendergast,[1,*] Gabor A. Somorjai,[3,4,*] and Ji Su[1,3,*]

1 The Molecular Foundry, Lawrence Berkeley National Laboratory, Berkeley, California 94720, United States.

2 State Key Laboratory of Physical Chemistry of Solid Surfaces, Collaborative Innovation Center of Chemistry for Energy Materials, and Department of Chemistry, College of Chemistry and Chemical Engineering, Xiamen University, Xiamen 361005, China.

3 Chemical Sciences Division, Lawrence Berkeley National Laboratory, Berkeley, California 94720, United States.

4 Department of Chemistry, University of California-Berkeley, Berkeley, California 94720, United States

5 Advanced Light Source, Lawrence Berkeley National Laboratory, Berkeley, California 94720, United States.

6 Sandia National Laboratories, Livermore, California 94551, United States.

7 Materials Science and Engineering Department, University of California-Berkeley, Berkeley, California 94720, United States.

† These authors contributed equally to this work.

*Email: dgprendergast@lbl.gov, somorjai@berkeley.edu, and jisu@lbl.gov

Contents

**Supplementary Methods**	4-6
**Catalysts preparation**	4
**In Situ DRIFTS study**	5
**X-ray absorption spectroscopy (XAS) measurements.**	5
**Ambient-Pressure X-ray Photoelectron Spectroscopy (APXPS) measurements.**	5
**Computational Details**	5-6
**Supplementary Tables**	7-8
**Supplementary Table 1** The best-fit EXAFS parameters of various samples.	7
**Supplementary Table 2** The start temperature of cyclohexane dehydrogenation and cyclohexane absorption on different catalysts.	7
**Supplementary Table 3** Proportions of Ce^{3+} and Ce^{4+} species in different samples (Standard Deviation: 2.1%).	8
**Supplementary Table 4** Partial atomic charges (in a.u.) obtained from Bader charge analysis for CeO_2, CeO_2_1V_O, Pt_1/CeO_2, and Pt_1/CeO_2_1V_O slabs with and without cyclohexane. Calculations performed using the PBE-D3(BJ)+U (*U* = 4.5 eV on Ce) method.	8
**Supplementary Figures**	9-
**Supplementary Figure 1** TEM images of (a) as-prepared CeO_2 nanorods, (b) single-site Pt_1/CeO_2 and (c) their corresponding XRD.	9
**Supplementary Figure 2** Schematic of cyclohexane dehydrogenation reactor.	9
**Supplementary Figure 3** TEM images of (a) 1%-2.5 nm Pt/CeO_2, (b)1%-7.0 nm Pt/CeO_2 and (c) their corresponding XRD.	10
**Supplementary Figure 4** Cyclic catalytic experiment of Pt_1/CeO_2 catalyst in cyclohexane dehydrogenation at different temperatures.	10
**Supplementary Figure 5** (a) Normalized XANES spectra, (b) k^3-weighted Fourier transform EXAFS spectra and (c) XAFS spectra of Pt_1/CeO_2, Pt_1/CeO_2 after a long time of cyclohexane dehydrogenation (Pt_1/CeO_2-D), Pt_1/CeO_2 after benzene hydrogenation (Pt_1/CeO_2-H), Pt_1/CeO_2-commercial, Pt_1/CeO_2 after cyclohexane dehydrogenation at 400 ^oC, PtO_2 and bulk Pt foil at the Pt L_3-edge. The hollow points are fitting results.	11
**Supplementary Figure 6** Turnover frequency (TOF) of hydrogen production per Pt site of Pt_1/CeO_2 of cyclohexane at different temperatures.	11
**Supplementary Figure 7** Comparison of H_2 evolution rates (calculated based on cyclohexane or MCH conversion) for Pt_1/CeO_2 and other reported Pt-based catalysts (References are cited in the Supplementary References).	12
**Supplementary Figure 8** Comparison of Pt_1/CeO_2 with 5% Pt/Al_2O_3 commercial catalyst at different temperatures.	12
**Supplementary Figure 9** In situ diffuse reflectance infrared Fourier transform spectroscopy (DRIFTS) following cyclohexane desorption on single-site catalysts at different times after stopping cyclohexane feed: (a) as-prepared CeO_2 support, (b) single-site Pt_1/CeO_2, (c) as prepared Al_2O_3 support, (d) single-site Pt_1/Al_2O_3, (e) commercial CeO_2 powders, and (f) isolated single Pt sites on commercial CeO_2 powders. (The peaks around 2930 cm^{-1} and 2852 cm^{-1} are the signals of cyclohexane absorption on support.)	13
**Supplementary Figure 10** (a) TEM images and (b) XRD of commercial CeO_2 particles.	13
**Supplementary Figure 11** XPS spectra and corresponding fitting curves of Ce 3*d* in (a) CeO_2, (b) CeO_2-commercial, (c) Pt_1/CeO_2 and (d) Pt_1/CeO_2-commercial and their deviation. The green peaks are attributed to Ce^{3+} species (881.0, 883.4, 899.3 and 903.1 eV) while orange peaks are attributed to Ce^{4+} species (882.2, 888.4, 898.1, 900.7, 907.3 and 916.7 eV).	14
**Supplementary Figure 12** Normalized XANES spectra of Ce $M_{4,5}$ edge in (a) CeO_2-prepared, Pt_1/CeO_2 and Pt_1/CeO_2 absorbed cyclohexane (Pt_1/CeO_2-cyclohexane) and (b) CeO_2-commercial, Pt_1/CeO_2-commercial and Pt_1/CeO_2-commercial absorbed cyclohexane (Pt_1/CeO_2-commercial-cyclohexane).	14

**Supplementary Figure 13** Catalytic benzene hydrogenation performance of Pt_1/CeO_2 catalyst with different (a) hydrogen pressure, (b) different time, (c) different benzene content and (d) different temperature. The error bars are defined as standard deviation of three experiments.	15
**Supplementary Figure 14** Catalytic performance of methylcyclohexane dehydrogenation and toluene hydrogenation over Pt_1/CeO_2 catalysts.	15
**Supplementary Figure 15** An oxygen vacancy V_O on the CeO_2 (111) surface (approximate center of each plot, surrounded by distorted hexagon). Color code – Ce^{4+}: light blue and dark blue; Ce^{3+}: turquoise; Pt: gray; O: red; H: white. The polyhedral faces are semi-transparent; therefore, we see a dark blue color for surface Ce^{4+} ions and a light blue color for sub-surface Ce^{4+} ions. (a) For pure CeO_2, V_O donates two electrons to form two Ce^{3+} small polarons. (b) Pt_{Ce} substitution at the surface with a neighboring V_O accepts both electrons to form Pt^{2+} with the familiar PtO_4 planar geometry (breaking two Pt-O bonds, by contrast with surface Ce atoms). (c) Two chemisorbed H bound to surface O, resulting in the formation of two Ce^{3+}.	16
**Supplementary Figure 16** Local bonding around the single-atom Pt site with and without an O vacancy: (a) 6-fold O coordination of Pt^{4+} instead of 7-folder for the pristine surface without an O vacancy; (b) 4-fold O coordination of Pt^{2+} with an O vacancy (colors as in Supplementary Figure 14).	16
**Supplementary Figure 17** Local bonding of organic intermediates around the single-atom Pt site: (a) Pt–C bonding (Pt^{3+}) for a chemisorbed intermediate (C_6H_{11}) following single H abstraction with simultaneous electron reduction of two Ce^{4+} to two Ce^{3+}; (b) C=C double-bond binding of C_6H_{10} to Pt^{2+} (reduced by conversions of Ce^{3+} to Ce^{4+}), with rotation of the Pt^{2+} bonding plane (gray polyhedron) to meet the double-bond, and creation of an additional Ce^{3+} as the H^+ combines with surface oxygen to make a hydroxyl; (c) A stable physiosorbed state of cyclohexa-1,3-diene and three chemisorbed H (with accompanying Ce^{3+}), designated as physisorption due to the large nearest Pt–H distance (2.38 Å) (colors as in Supplementary Figure 14).	16
**Supplementary Figure 18** Local bonding around the single-atom Pt site during H reactions: (a) H_2 physisorption perpendicular to the PtO_4 plane; (b) H_2 dissociation with the formation of a covalent Pt–H bond (Pt^{3+}) and restoration of 6-fold coordination in addition to the formation of two Ce^{3+}; (c) Two chemisorbed H^+ bound as OH^- and two Ce^{3+} created as a result of it (colors as in Supplementary Figure 14).	17
**Supplementary Figure 19** Reaction energies relative to the starting reactants (gas phase cyclohexane and the bare substrate) for each of the intermediate steps indicated in Fig. 5 in the manuscript. The inset includes energies for dehydrogenation of the catalyst surface (if moving from left to right through the intermediates) or, alternatively, hydrogenation prior to benzene adsorption (with respect to gas phase H_2 and the bare substrate) as in the interior of Fig. 5.	17
**Supplementary Figure 20** Localized density of states (LDOS)/projected density of states (PDOS) plots of Ce and Pt ions in four systems, (a) CeO_2_slab, (b) CeO_2_1V_O slab, (c) Pt_1/CeO_2_slab, and (d) Pt_1/CeO_2_1V_O slab, displaying their d and f bands.	18-20
**Supplementary References**	21-22

Supplementary Methods

Catalysts preparation

Chemicals and materials. Cerium (III) nitrate hexahydrate ($Ce(NO_3)_3·6H_2O$, 99%), sodium hydroxide (NaOH, 97%), ascorbic acid (AA, $C_6H_8O_6$, 99%), chloroplatinate (IV) hexahydrate ($H_2PtCl_6·6H_2O$, 99.9%), polyvinylpyrrolidone (PVP), Cerium oxide (CeO_2, 99.5%), cyclohexane (C_6H_{12}, 99.5%), methylcyclohexane (C_7H_{14}, 99%), benzene (C_6H_6, 99.9%), toluene ($C_6H_5CH_3$, 99.8%), heptane ($CH_3(CH_2)_5CH_3$, 99%) and ethylene glycol (C_2H_6O, 99.8%) were purchased from Sigma-Aldrich. All reagents were used without further purification.

Synthesis of porous CeO_2. Porous CeO_2 nanorods were synthesized according to previous reports.[1] 4 mmol $Ce(NO_3)_3·6H_2O$ (1.736 g) and 0.48 mol NaOH (19.2 g) were dissolved in 80 ml distilled water. After stirring for 30 mins at room temperature, the mixed solution was transferred to a 100 ml Teflon-lined stainless-steel autoclave and kept at 100 ^oC for 12 h. The products were collected by centrifugation and washed with distilled water several times. After drying in vacuum at 60 ^oC for 12 h, the products were calcined at 400 ^oC in air for 1 h.

Synthesis of 2.5 nm Pt nanoparticles and 7.0 nm Pt nanoparticles. Pt nanoparticles with 2.5 and 7.0 nm sizes were synthesized according to the method reported in literature. For the synthesis of 2.5 nm Pt nanoparticles, 133 mg PVP and 20 mL of $H_2PtCl_6·6H_2O$ aqueous solution (6.0 mM) were dissolved in 180 mL ethanol. Then the solution was refluxed for 3 h at 90 ^oC. After washing with methanol and acetone several times, the products were dried and then dispersed in water, and the concentration was kept at 5 mg/mL. For the synthesis of 7.0 nm Pt nanoparticles, 3 mL ethylene glycol solution of PVP (0.375 M) and ethylene glycol solution of $H_2PtCl_6·6H_2O$ (0.0625 M) were alternately added to 2.5 ml boiling ethylene glycol every 30 s. The mixture was further refluxed for 10 min, and the particles were collected by centrifugation and redispersed in water with a concentration of 5 mg/mL.[2]

Synthesis of 2.5 nm Pt/CeO_2 and 7.0 nm Pt/CeO_2. 500 mg CeO_2 was dispersed in 20 mL distilled water and to it was added 1 mL of 5 mg/mL Pt nanoparticle solution. After ultrasonic treatment for 1 h and stirring overnight, the particles were dried in vacuum at 100 ^oC. The products are named as 2.5 nm Pt/CeO_2 and 7.0 nm Pt/CeO_2. The prepared Pt/CeO_2 composite was further processed with UV-ozone treatment to remove the capped PVP layer.

Synthesis of single-site Pt_1/Al_2O_3 catalyst. Pt_1/Al_2O_3 catalyst was synthesized according to a previous report.[3] In a typical synthesis, 2.1 g Pluronic P123 was dissolved in 20 mL ethanol at room temperature. Then, 67% 3.2 mL nitric acid and 4.08 g aluminum isopropoxide were also dissolved in 20 mL ethanol. The two solutions were mixed under vigorous stirring. Next, 0.2 wt% H_2PtCl_6 dissolved in ethanol (0.0193 mol/L) was added to the mixture being stirred. The solution was stirred at room temperature for 48 h and then placed in an oven at 60 °C for ethanol evaporation for 72 h. The final gel was calcined at 400 °C for 4 h at a heating rate of 1 °C /min, and then reduced in 5% H_2/N_2 at 400 °C for 1 h at a heating rate of 5 °C/min.

In Situ DRIFTS study

Diffuse reflectance infrared Fourier transform spectroscopy (DRIFTS) was recorded on a Thermo Nicolet 6700 equipped with an in situ reaction chamber, which is covered by KBr filters. The sample was in situ activated at 300 ˚C for 60 mins under 50 mL/min N_2 flow. Then 30 mL/min N_2 was purged into pure cyclohexane for 1 min, and the vapor phase was carried into a DRIFT cell. Then pure 30 ml/min N_2 was flown in to remove the gas phase cyclohexane.

X-ray absorption spectroscopy (XAS) measurements

Ce M-edge X-ray absorption spectra were measured at beamline 7.3.1 in Advanced Light Source, Lawrence Berkeley National Laboratory. The Ce M-edge XAS spectra were recorded in total electron yield with 0.3 eV energy resolution. The measurements of Pt $\boldsymbol{L}_3$-edge XAS spectra including X-ray absorption near edge structure (XANES) and extended X-ray absorption fine structure (EXAFS) were performed at the TPS 44A beamline of the National Synchrotron Radiation Research Center (NSRRC) Taiwan. The data were collected in fluorescence mode by using 7-element silicon drift detector and the standard Pt-foil was used as reference for the energy calibration. The data were processed according to standard procedures using Demeter program package.[4]

Ambient-Pressure X-ray Photoelectron Spectroscopy (APXPS) measurements

APXPS experiments were performed at beamline 9.3.2 in Advanced Light Source, Lawrence Berkeley National Laboratory. The 9.3.2 end station is equipped with a differentially pumped VG Scienta hemispherical analyzer with an 800 µm diameter aperture. The bending magnet spherical grating monochromator beamline has a usable energy range of 200–900 eV capable of ΔE/E resolving power better than 1/1000. Pt_1/CeO_2 powders were dropped on gold foil. The Ce 4d, Pt 4f, O 1s, C 1s and Au 4f spectra of samples were first collected at different temperatures (room temperature and reaction temperature of 350 ^{o}C). After feeding cyclohexane gas, the spectra of Ce 4d, Pt 4f, O 1s, C 1s and Au 4f were examined both at room temperature and 350 ^{o}C in 0.1 Torr cyclohexane.

Computational Details

Systems. The unit cell of the cubic crystal structure of CeO_2 consisting of 12 atoms was optimized. An extended slab was carved from the optimized cubic structure, exposing the (111) surface – no other surfaces were considered for this study. The slab has three monolayers of CeO_2 and 15 Å vacuum above it. CeO_2 slabs with and without Pt doping and with and without an oxygen vacancy on the surface were used in the calculations.

Methods. All calculations were performed using density functional theory (DFT) with periodic boundary conditions as implemented in the *Vienna Ab Initio Simulation Package* (*VASP*).[5,6] The DFT calculations employ the PBE[7] generalize-gradient exchange-correlation functional with and without Hubbard (+U) corrections, along with Becke-Johnson damping for dispersion corrections,[8] referred to as PBE-D3(BJ)+U. The on-site Hubbard *U* correction[9,10] of 4.5 eV is applied only to the *f* electrons of Ce, where the choice of +U value is based on the recommendation in Supplementary References 11.

Cubic structure calculations: For calculations on the cubic crystal structure of CeO_2, the nuclear positions, the cell shape, and the cell volume of the unit cell were optimized. Plane-wave basis sets with a cut-off energy of 600 eV and the PBE projector-augmented-wave (PAW) potentials that come with the *VASP* package were used to represent the electronic structure of the crystal.[12, 13] A *k*-point mesh of 6x6x6 is used to sample the Brillouin zone. For convergence criteria, 10^{-6} eV or less is used for the self-consistent field (SCF) energy and -10^{-3} eV/Å is used for the forces.

Slab calculations: For calculations on the slabs, the nuclear positions were optimized, but the cell shape and cell volume were kept fixed. A cutoff energy of 400 eV along with the PBE PAW potentials were used to represent the electronic structure of the slab. A *k*-point mesh of 2x2x1 is used to sample the Brillouin zone. For convergence criteria, 10^{-6} eV or less is used for the SCF energy and -10^{-3} eV/Å is used for the forces.

The Pt_1/CeO_2 catalytic surface considered here, has one surface oxygen vacancy (the system is referred to as $Pt_1/CeO_2\_1V_O$ henceforth). To obtain $Pt_1/CeO_2\_1V_O$, we start with a clean CeO_2 slab, replace a surface Ce atom by a Pt atom, which leads to the Pt_1/CeO_2 slab. Then we add an O vacancy at the surface in the first coordination sphere of the Pt atom, leading to the $Pt_1/CeO_2\_1V_O$ slab. The CeO_2 (111) slab comprises 48 Ce and 96 O atoms. The Pt_1/CeO_2 slab has one Pt, 47 Ce, and 96 O atoms when there is no O vacancy. The $Pt_1/CeO_2\_1V_O$ slab that has one surface O vacancy has one Pt, 47 Ce, and 95 O atoms.

We report relative energies and oxidation states of various steps of the reaction cycle. The relative energies of each step are calculated with respect to the infinitely separated reactants, which are, cyclohexane and the $Pt_1/CeO_2\_1V_O$ slab. To confirm the oxidation state of Ce, for example, to check for Ce^{3+}, we examine the local electronic spins for each atom. Reduced Ce^{3+} localizes an unpaired 4f electron .

Charge analysis. Bader charge analysis[14-17] was performed on the slabs to determine partial atomic charges on each atom and to see how the charges change upon Pt doping, upon incorporation of an oxygen vacancy on the ceria slab, or during the progress of the reaction. Supplementary Table 4 gives Bader charges for some of the initial systems of the reaction cycle.

Oxidation state analysis. The electronic density of states (DOS) for ceria slab is indicative of the Ce^{4+} oxidation state, with electronic configuration $[Xe]6s^0 4f^0$. The lowest energy orbitals in the unoccupied DOS define a narrow band of highly localized Ce 4*f* orbitals (see Supplementary Figure 20 (a)). The presence of each oxygen vacancy in ceria frees two valence electrons which can fill two orbitals in this narrow band and lead to the formation of two small polarons – these electrons localize on two separate Ce sites, due to induced elongation of the apical Ce–O bond lengths local to their respective Ce ions, which breaks translational symmetry, and effectively reduces them (nominally from +4 to +3). This results in a shift of the conduction band toward the Fermi level (compare Supplementary Figure 20 (a) and (b)). With a Pt_{Ce} substitution, as in the Pt_1/CeO_2 system (with no O vacancy), the formal oxidation state of both Pt and Ce is +4. However, when an O vacancy is present within the first coordination sphere of the substituted Pt, the available lower energy and more delocalized Pt 5d orbitals are preferentially filled, with a formal reduction of Pt from +4 to +2 and no evident reduction of Ce (as seen in the $Pt_1/CeO_2\_1V_O$ slab, Supplementary Figure 20 (c)). The local O coordination geometry around the reduced Pt adopts a rhomboid planar structure, forming the familiar PtO_4 group of this 2+ oxidation state due to elongation or breaking of two of the original six Pt–O bonds (bulk O coordination in ceria is 8; 7 at the (111) surface; 6 with an O vacancy).

Supplementary Tables

Supplementary Table 1 The best-fit EXAFS parameters of various samples.

Sample	Scattering Path	N	R (Å)	σ^2 (Å^2)	Rf
Pt foil	Pt-Pt bond	12	2.76	0.004	0.001
PtO_2	Pt-O bond	6	2.02	0.002	0.003
Pt_1/CeO_2	Pt-O bond	5.8	1.99	0.002	0.002
Pt_1/CeO_2-D	Pt-O bond	5.7	1.99	0.002	0.001
Pt_1/CeO_2-H	Pt-O bond	3.7	2.01	0.004	0.003
Pt_1/CeO_2-commercial	Pt-O bond	5.9	2.00	0.003	0.002
Pt_1/CeO_2-400 ^oC	---	---	---	---	---

The curve fitting was conducted in R-space with the fitting range (ΔR) and the R-factor (Rf) of fit indicated in the table, where N is the coordination number, R is the distance between absorber and backscatter atoms, σ^2 is the Debye–Waller factor value, ΔR is the data range for fitting in R-space, and Rf is the R-factor characterizing the goodness of fit. Error bounds (accuracies) characterizing the structural parameters obtained by EXAFS data analysis are estimated to be as follows: N, ±20%; R, ±1%; and σ^2, ±20%.

Supplementary Table 2 The start temperature of cyclohexane dehydrogenation and cyclohexane adsorption on different catalysts.

Sample name	$Temperature_{start}$[1]	Cyclohexane adsorption[2]
0.15%-Pt_1/CeO_2	100 ^oC	Yes
1%-2.5 nm Pt/CeO_2-UV-treatment	450 ^oC	Yes
1%-7.0 nm Pt/CeO_2-UV-treatment	450 ^oC	Yes
1%-2.5 nm Pt/CeO_2-cal-reduction	450 ^oC	Yes
1%-2.5 nm Pt/CeO_2-AA-cal	400 ^oC	Yes
10%-2.5 nm Pt/CeO_2	400 ^oC	Yes
1%-2.5 nm Pt/CeO_2-AA	450 ^oC	Yes
Pt_1/CeO_2-commerical	200 ^oC	Yes[3]
0.1%-Pt_1/Al_2O_3	300 ^oC	No
5%-2.5 nm Pt/Al_2O_3	300 ^oC	No
5%-Pt/Al_2O_3-commercial	150 ^oC[4]	No

[1]The start conversion temperature was tested by flow-reactor equipped with GC. Reaction conditions: 100 mg catalyst mixed with 500 mg sand; Gas flow: N_2 30 mL/min; Cyclohexane feeding rate: 3 mL/h.

[2]The DRIFT study of cyclohexane adsorption was carried out at room temperature. Sample was in situ activated at 300 ^oC for 60 min under 50 mL/min N_2 flow. 30 mL/min N_2 was then purged into cyclohexane for 1 min, and the vapor phase was carried into DRIFT cell. Then pure 30 mL/min N_2 was flown in to remove the gas phase cyclohexane.

[3]For pure commercial CeO_2 there is no adsorption of cyclohexane, and after treating with AA the cyclohexane adsorption was detected by DRIFTS.

[4]Cyclohexane started to dehydrogenate at 150 ^oC on 5%-Pt/Al_2O_3 commercial catalyst, however, at the same reaction temperature, the single-site Pt_1/CeO_2 catalyst gave 100 times TOF compared to the Pt/Al_2O_3 catalyst.

Supplementary Table 3 Proportions of Ce^{3+} and Ce^{4+} species in different samples (Standard Deviation: 2.1%).

Sample	Ce^{3+} species (%)	Ce^{4+} species (%)
CeO_2	22.6	77.4
CeO_2-commercial	12.7	88.3
Pt_1/CeO_2	29.4	70.6
Pt_1/CeO_2-commercial	19.7	81.3

The proportion of Ce^{3+} species, P(Ce^{3+}), was calculated using the following equation, of which the area is the fitting area in XPS:

Supplementary Equation 1: $\text{Proportion}\ (Ce^{3+}) = \frac{area\ (Ce^{3+})}{area\ (Ce^{3+}) + area\ (Ce^{4+})} \times 100\%$

Supplementary Table 4 Partial atomic charges (in a.u.) obtained from Bader charge analysis for CeO_2, CeO_2_$1V_O$, Pt_1/CeO_2, and Pt_1/CeO_2_$1V_O$ slabs with and without cyclohexane. Calculations performed using the PBE-D3(BJ)+U (U = 4.5 eV on Ce) method.

**System**[a]	**Charge (avg, min, max)**[b]		**Charge**[c]	
	**Ce^{4+}**	**O**	**Pt**	**Ce^{3+}**
**No O vacancy**				
CeO_2_slab	2.40, 2.39, 2.41	-1.20, -1.23, -1.18	–	–
CeO2_slab—cyclohexane	2.40, 2.39, 2.41	-1.20, -1.23, -1.18	–	–
Pt_1/CeO_2_slab	2.28, 2.24, 2.30	-1.13, -0.97, -1.18	1.37	–
Pt_1/CeO_2_slab—cyclohexane	2.39, 2.36, 2.41	-1.19, -1.01, -1.24	1.35	–
**One O vacancy**				
CeO_2_slab_$1V_O$	2.39, 2.32, 2.43	-1.20, -1.23, -1.18	–	2.14, 2.14
CeO_2_slab_$1V_O$—cyclohexane	2.39, 2.32, 2.43	-1.20, -1.26, -1.16	–	2.13, 2.12
Pt_1/CeO_2_slab_$1V_O$	2.28, 2.21, 2.30	-1.14, -1.18, -1.02	0.86	–
Pt_1/CeO_2_slab_$1V_O$—cyclohexane	2.39, 2.32, 2.41	-1.19, -1.23, -1.06	0.84	–

[a] $1V_O$ indicates one oxygen vacancy on the surface of the slab.

[b] The partial atomic charges for (nominal) Ce^{4+} and O is indicated using the average, minimum, and maximum values (avg, min, max).

[c] The specific charges of each Pt and Ce^{3+} are provided.

Supplementary Figures

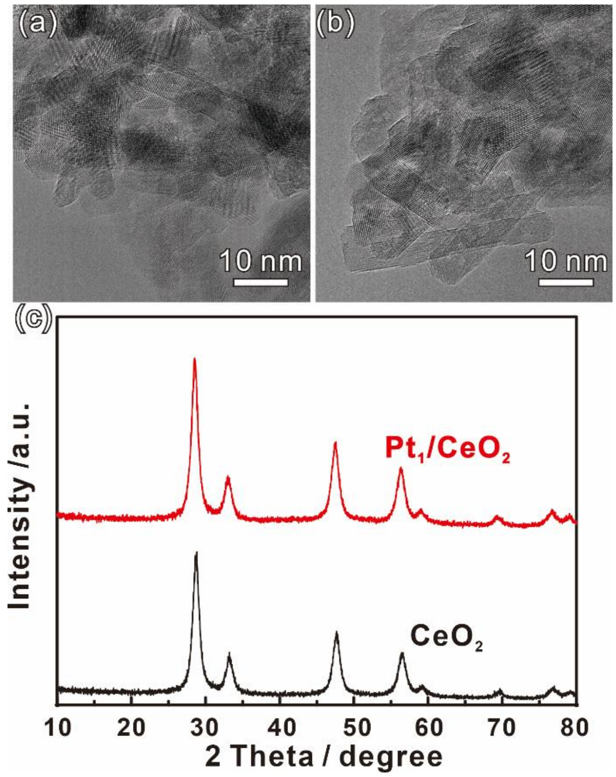

Supplementary Figure 1 TEM images of (a) as-prepared CeO_2 nanorods, (b) single-site Pt_1/CeO_2 and (c) their corresponding XRD.

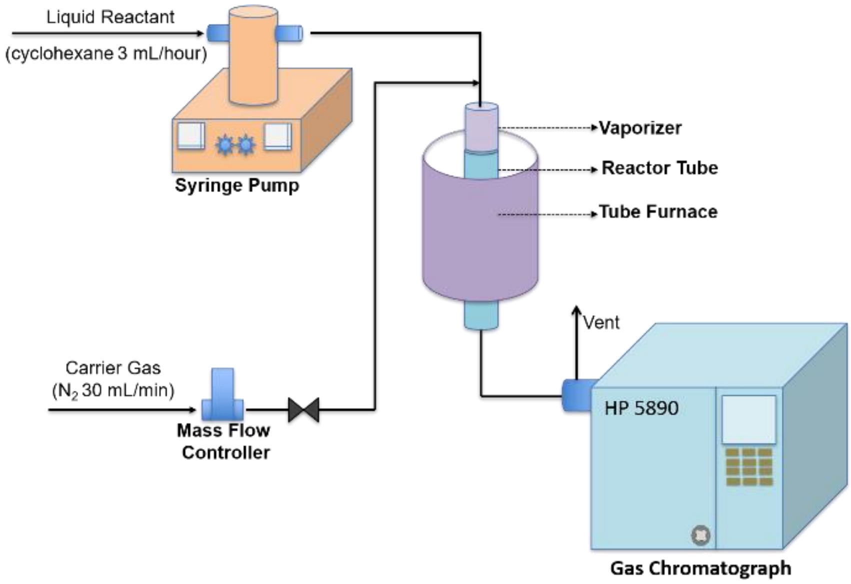

Supplementary Figure 2 Schematic of cyclohexane dehydrogenation reactor.

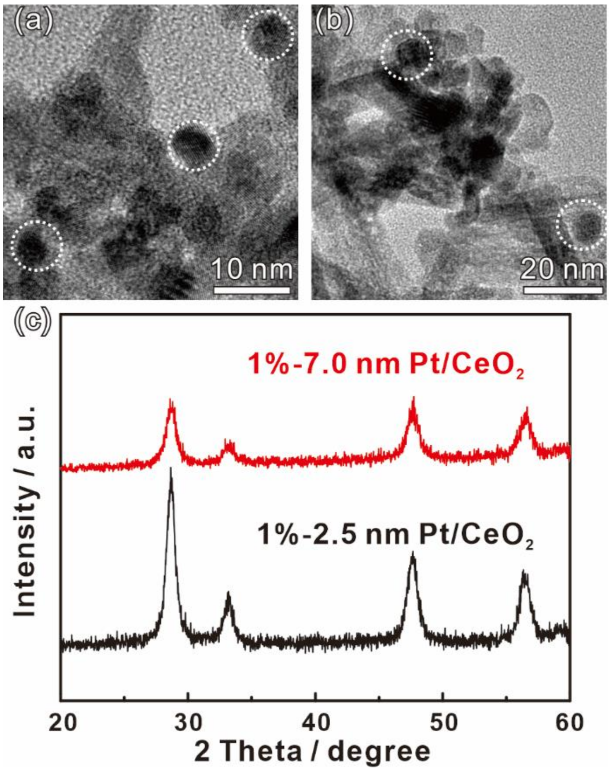

Supplementary Figure 3 TEM images of (a) 1%-2.5 nm Pt/CeO_2, (b) 1%-7.0 nm Pt/CeO_2 and (c) their corresponding XRD.

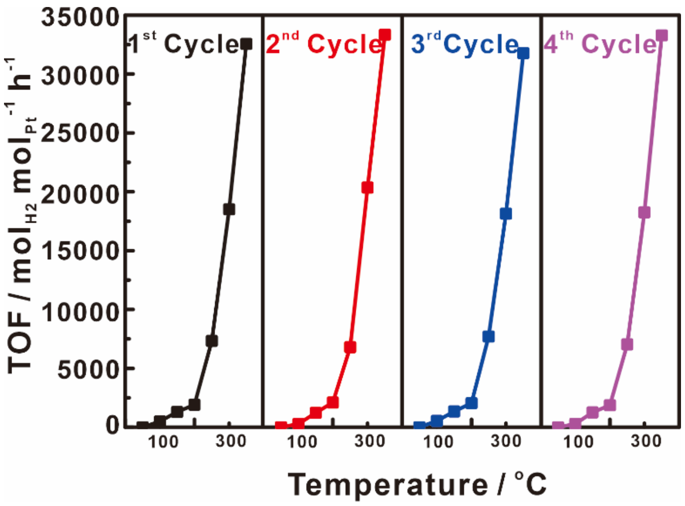

Supplementary Figure 4 Cyclic catalytic experiment of Pt_1/CeO_2 catalyst for cyclohexane dehydrogenation at different temperatures. Reaction conditions: 100 mg catalyst mixed with 500 mg sand; Gas flow: N_2 30ml/min; Cyclohexane feeding rate: 3 mL/h.

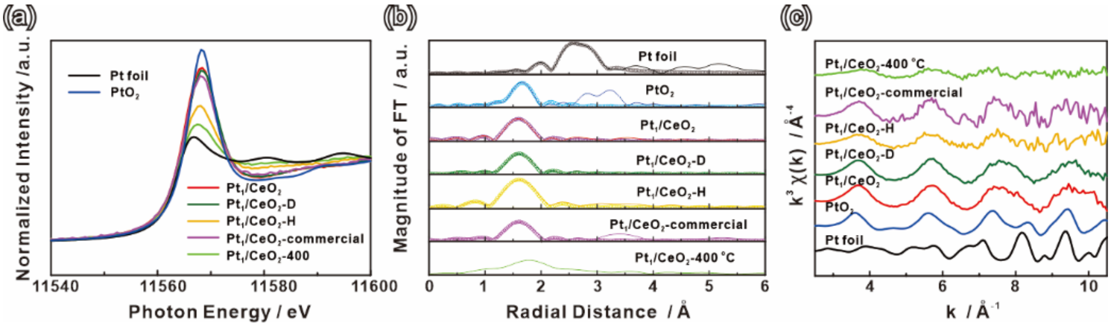

Supplementary Figure 5 (a) Normalized XANES spectra, (b) k^3-weighted Fourier transform EXAFS spectra and (c) XAFS spectra of Pt_1/CeO_2, Pt_1/CeO_2 after a long time of cyclohexane dehydrogenation (Pt_1/CeO_2-D), Pt_1/CeO_2 after benzene hydrogenation (Pt_1/CeO_2-H), Pt_1/CeO_2-commercial, Pt_1/CeO_2 after cyclohexane dehydrogenation at 400 °C, PtO_2 and bulk Pt foil at the Pt L_3-edge. The hollow points are fitting results.

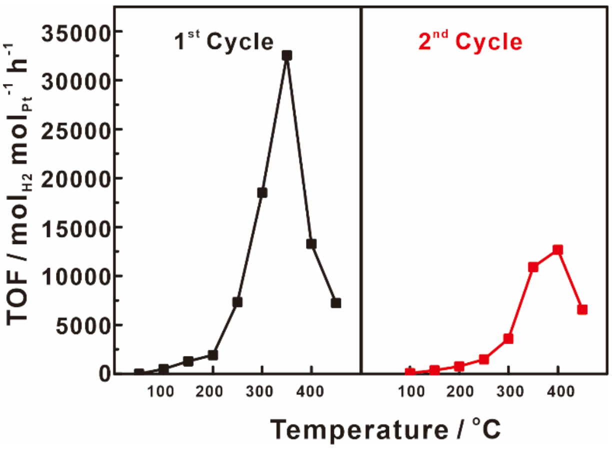

Supplementary Figure 6 Turnover frequency (TOF) of hydrogen production per Pt site of Pt_1/CeO_2 for cyclohexane dehydrogenation at different temperatures.

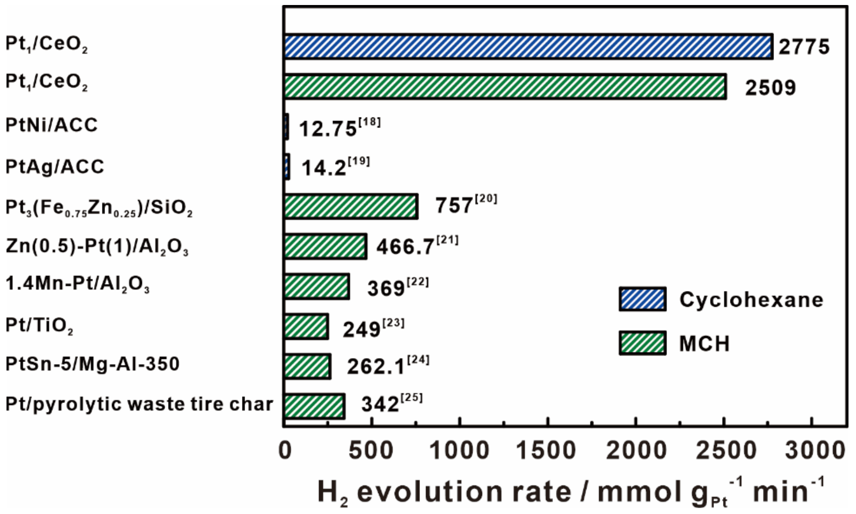

Supplementary Figure 7 Comparison of H_2 evolution rates (calculated based on cyclohexane or MCH conversion) for Pt_1/CeO_2 and other reported Pt-based catalysts (References are cited in the Supplementary References).

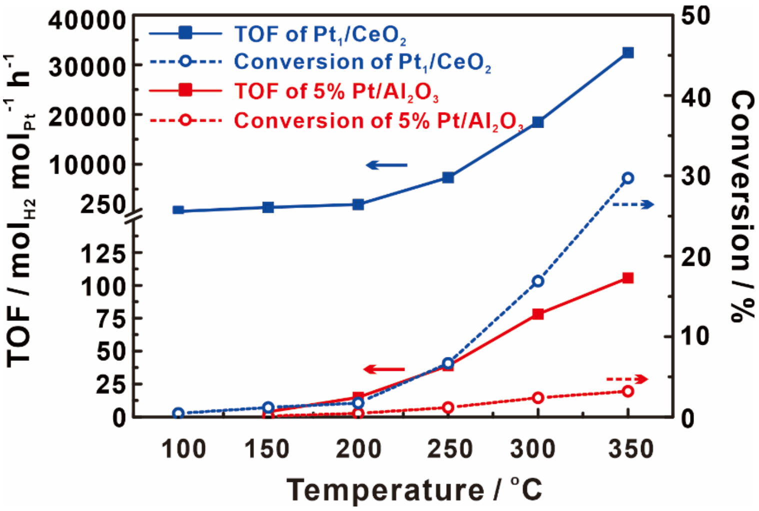

Supplementary Figure 8 Comparison of Pt_1/CeO_2 with 5% Pt/Al_2O_3 commercial catalyst at different temperatures. The solid line with square symbol is turnover frequency (TOF) and the dashed line with cycle symbol is conversion of cyclohexane.

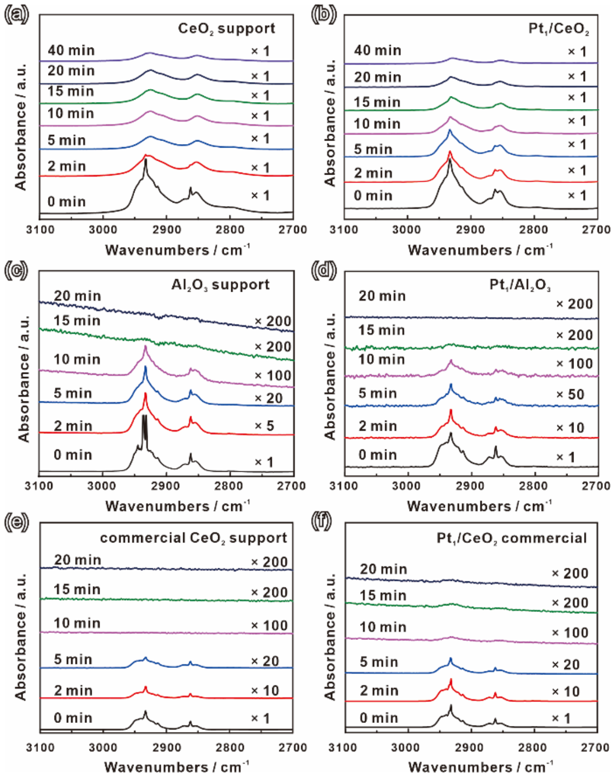

Supplementary Figure 9 In situ diffuse reflectance infrared Fourier transform spectroscopy (DRIFTS) following cyclohexane desorption on single-site catalysts at different times after stopping cyclohexane feed: (a) as-prepared CeO_2 support, (b) single-site Pt_1/CeO_2, (c) as prepared Al_2O_3 support, (d) single-site Pt_1/Al_2O_3, (e) commercial CeO_2 powders, and (f) isolated single Pt sites on commercial CeO_2 powders. (The peaks around 2930 cm^{-1} and 2852 cm^{-1} are the signals of cyclohexane absorption on the support.)

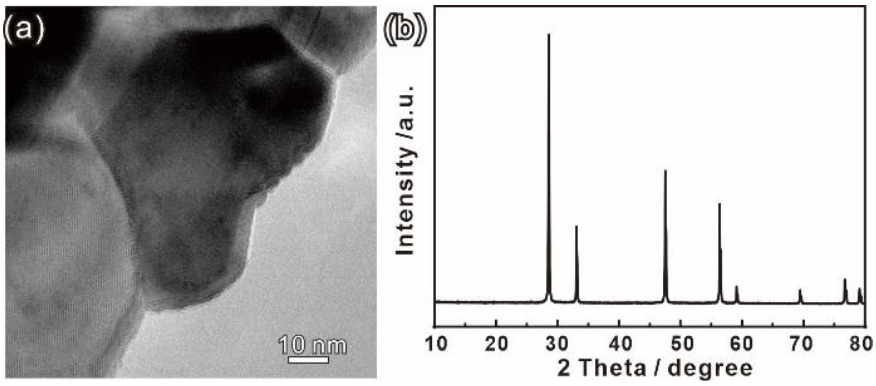

Supplementary Figure 10 (a) TEM images and (b) XRD of commercial CeO_2 particles.

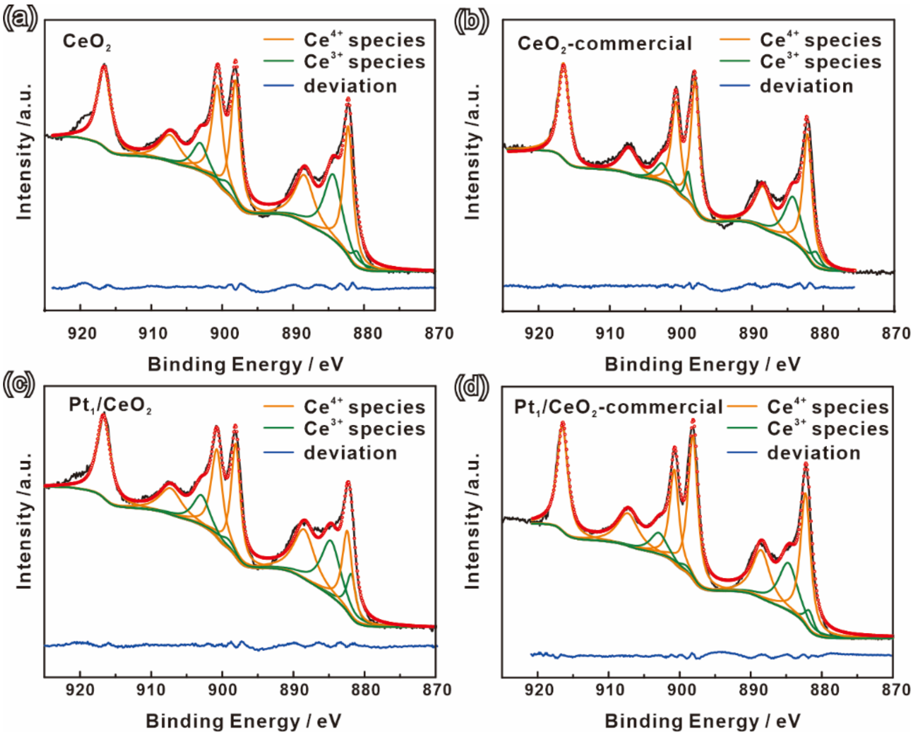

Supplementary Figure 11 XPS spectra and corresponding fitting curves of Ce 3*d* in (a) CeO_2, (b) CeO_2-commercial, (c) Pt_1/CeO_2 and (d) Pt_1/CeO_2-commercial and their deviation. The green peaks are attributed to Ce^{3+} species (881.0, 883.4, 899.3 and 903.1 eV) while the orange peaks are attributed to Ce^{4+} species (882.2, 888.4, 898.1, 900.7, 907.3 and 916.7 eV).

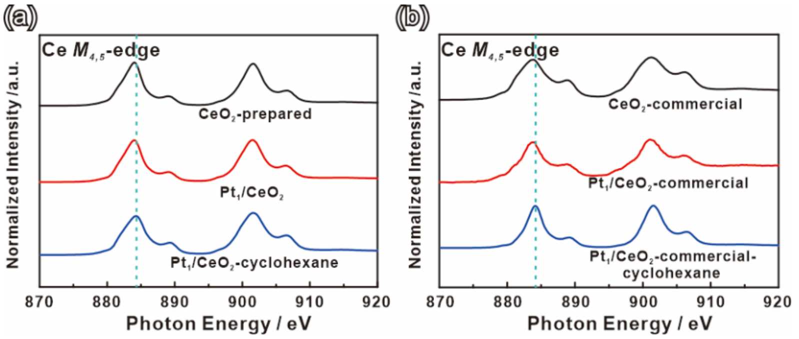

Supplementary Figure 12 Normalized XANES spectra of Ce $M_{4,5}$ edge in (a) CeO_2-prepared, Pt_1/CeO_2 and Pt_1/CeO_2 absorbed cyclohexane (Pt_1/CeO_2-cyclohexane) and (b) CeO_2-commercial, Pt_1/CeO_2-commercial and Pt_1/CeO_2-commercial absorbed cyclohexane (Pt_1/CeO_2-commercial-cyclohexane).

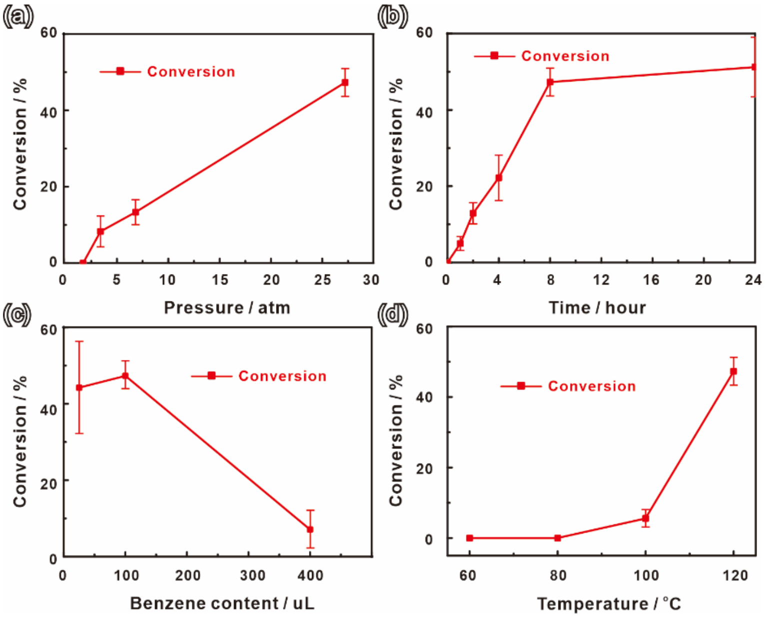

Supplementary Figure 13 Catalytic benzene hydrogenation performance of Pt_1/CeO_2 catalyst with (a) different hydrogen pressure, (b) different time, (c) different benzene content and (d) different temperature. The error bars are defined as standard deviation of three experiments.

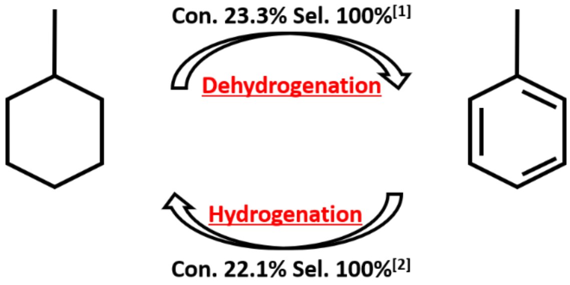

Supplementary Figure 14 Catalytic performance of methylcyclohexane dehydrogenation and toluene hydrogenation over Pt_1/CeO_2 catalysts.

[1]Dehydrogenation reaction conditions: 100 mg catalyst mixed with 500 mg sand; Gas flow: N_2 30 ml/min; Methylcyclohexane feeding rate: 3 mL/h; temperature: 350 ºC.
[2]Hydrogenation reaction conditions: 40 mg catalyst dispersed in 3 mL n-heptane; added 100 μL toluene under 27.22 atm hydrogen at 120 ºC and stirred for 12 h.

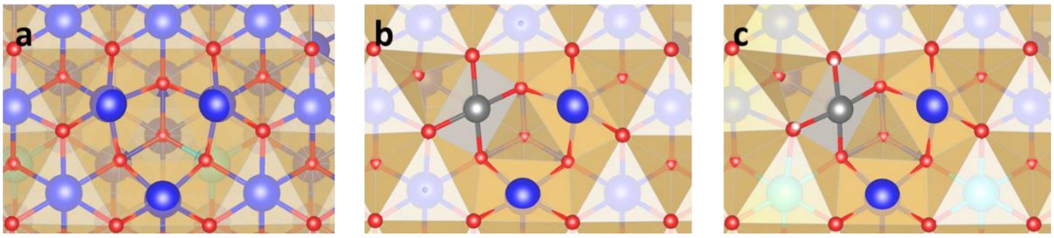

Supplementary Figure 15 An oxygen vacancy V_O on the CeO_2 (111) surface (approximate center of each plot, surrounded by distorted hexagon). Color code – Ce^{4+}: light blue and dark blue; Ce^{3+}: turquoise; Pt: gray; O: red; H: white. The polyhedral faces are semi-transparent; therefore, we see a dark blue color for surface Ce^{4+} ions and a light blue color for sub-surface Ce^{4+} ions. (a) For pure CeO_2, V_O donates two electrons to form two Ce^{3+} small polarons. (b) Pt_{Ce} substitution at the surface with a neighboring V_O accepts both electrons to form Pt^{2+} with the familiar PtO_4 planar geometry (breaking two Pt-O bonds, by contrast with surface Ce atoms). (c) Two chemisorbed H bound to surface O, resulting in the formation of two Ce^{3+}.

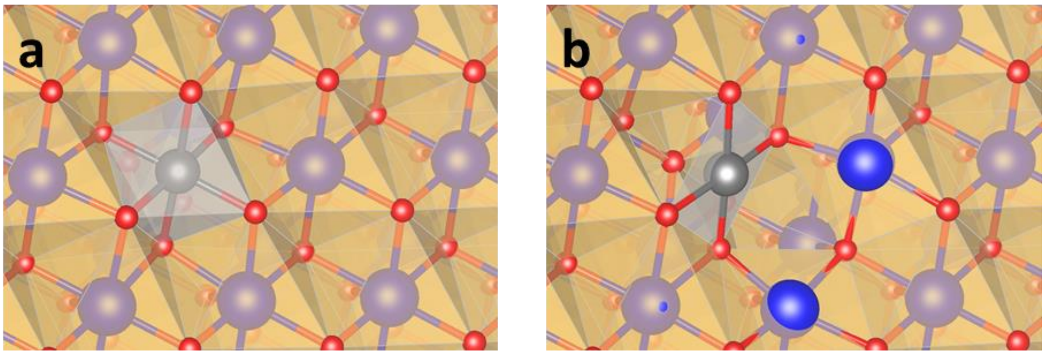

Supplementary Figure 16 Local bonding around the single-atom Pt site with and without an O vacancy: (a) 6-fold O coordination of Pt^{4+} instead of 7-folder for the pristine surface without an O vacancy; (b) 4-fold O coordination of Pt^{2+} with an O vacancy (colors as in Supplementary Figure 15).

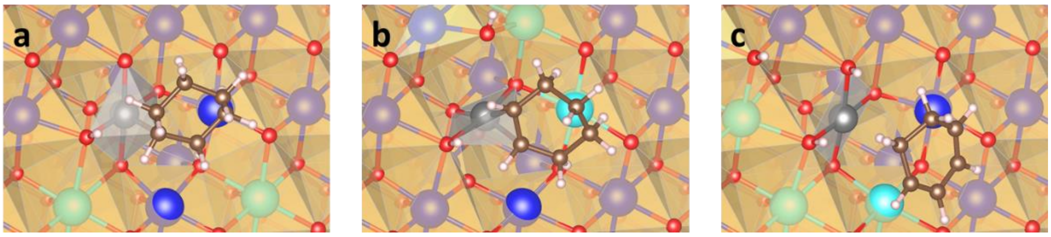

Supplementary Figure 17 Local bonding of organic intermediates around the single-atom Pt site: (a) Pt–C bonding (Pt^{3+}) for a chemisorbed intermediate (C_6H_{11}) following single H abstraction with simultaneous electron reduction of two Ce^{4+} to two Ce^{3+}; (b) C=C double-bond binding of C_6H_{10} to Pt^{2+} (reduced by conversions of Ce^{3+} to Ce^{4+}), with rotation of the Pt^{2+} bonding plane (gray polyhedron) to meet the double-bond, and creation of an additional Ce^{3+} as the H^+ combines with surface oxygen to make a hydroxyl; (c) A stable physiosorbed state of cyclohexa-1,3-diene and three chemisorbed H (with accompanying Ce^{3+}), designated as physisorption due to the large nearest Pt–H distance (2.38 Å) (colors as in Supplementary Figure 15).

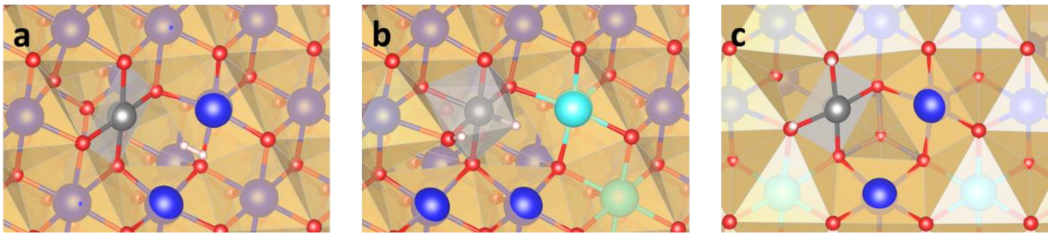

Supplementary Figure 18 Local bonding around the single-atom Pt site during H reactions: (a) H_2 physisorption perpendicular to the PtO_4 plane; (b) H_2 dissociation with the formation of a covalent Pt–H bond (Pt^{3+}) and restoration of 6-fold coordination in addition to the formation of two Ce^{3+}; (c) Two chemisorbed H^+ bound as OH^- and two Ce^{3+} created as a result of it (colors as in Supplementary Figure 15).

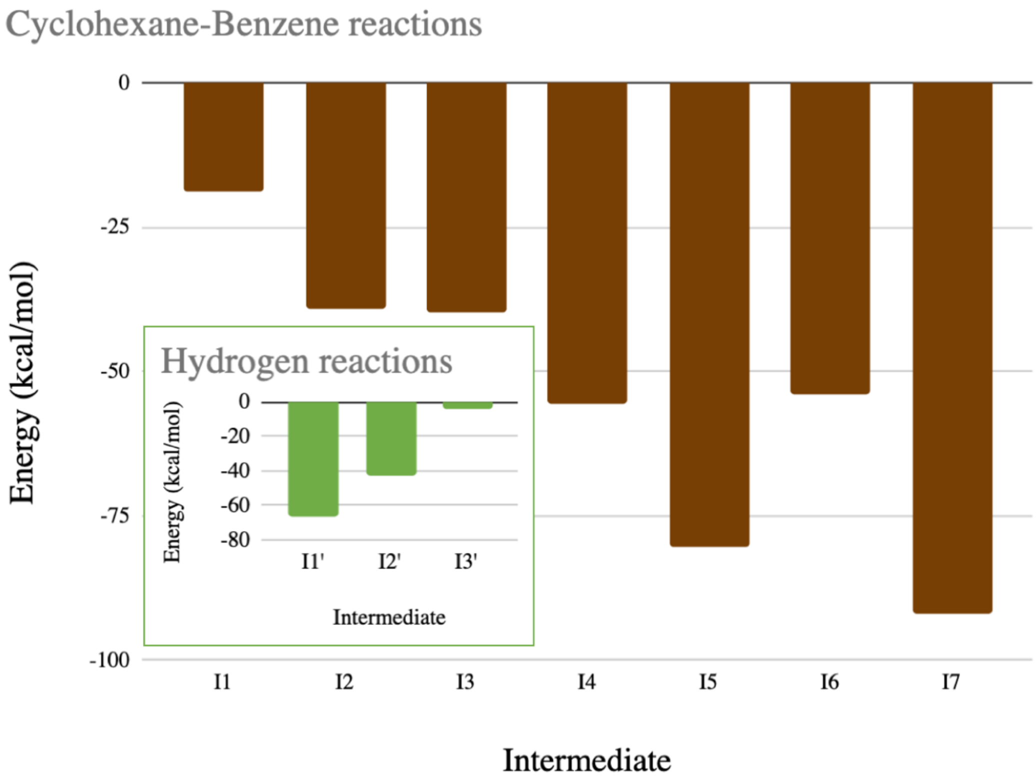

Supplementary Figure 19 Reaction energies relative to the starting reactants (gas phase cyclohexane and the bare substrate) for each of the intermediate steps indicated in Fig. 5 in the manuscript. The inset includes energies for dehydrogenation of the catalyst surface (if moving from left to right through the intermediates) or, alternatively, hydrogenation prior to benzene adsorption (with respect to gas phase H_2 and the bare substrate) as in the interior of Fig. 5.

(a) CeO_2 slab

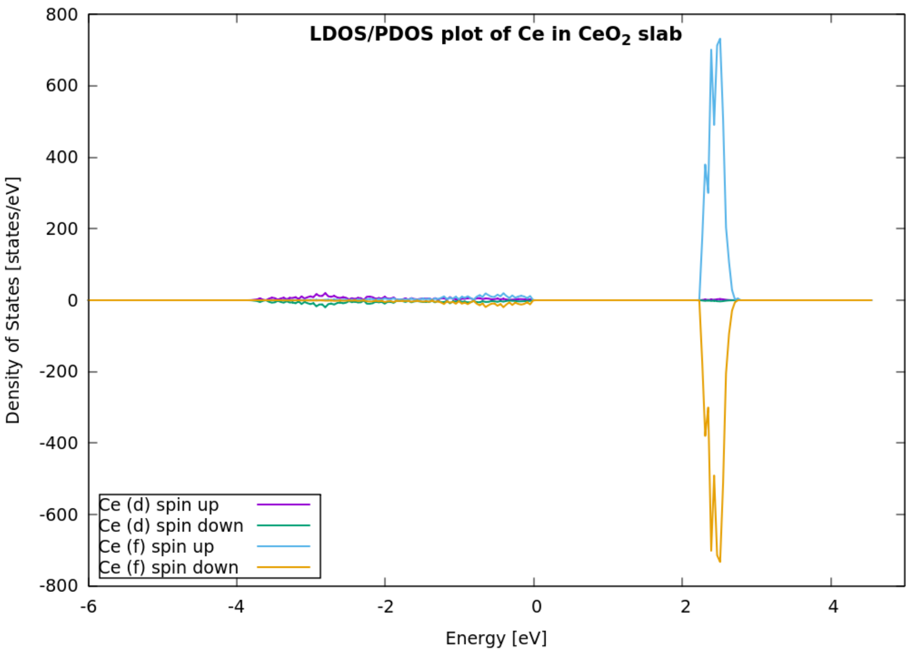

(b) CeO_2_$1V_O$ slab

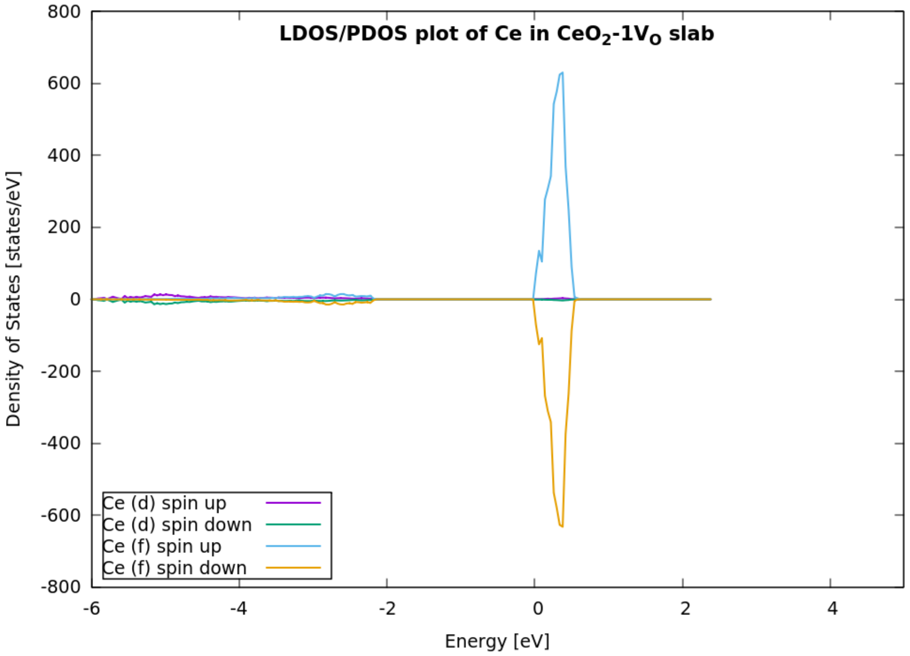

(c) Pt_1/CeO_2 slab

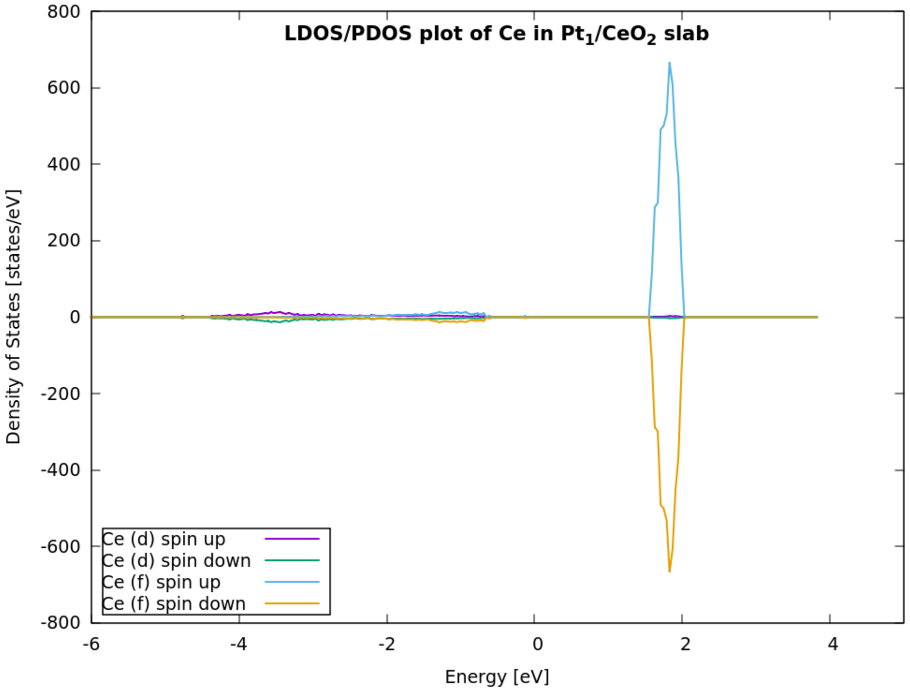

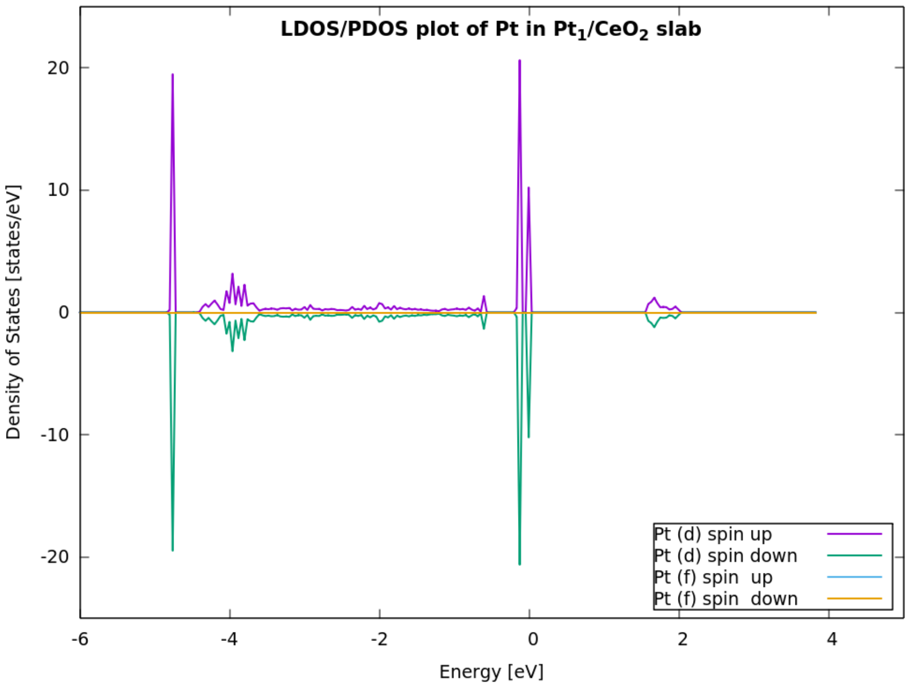

(d) Pt_1/CeO_2 _$1V_O$ slab

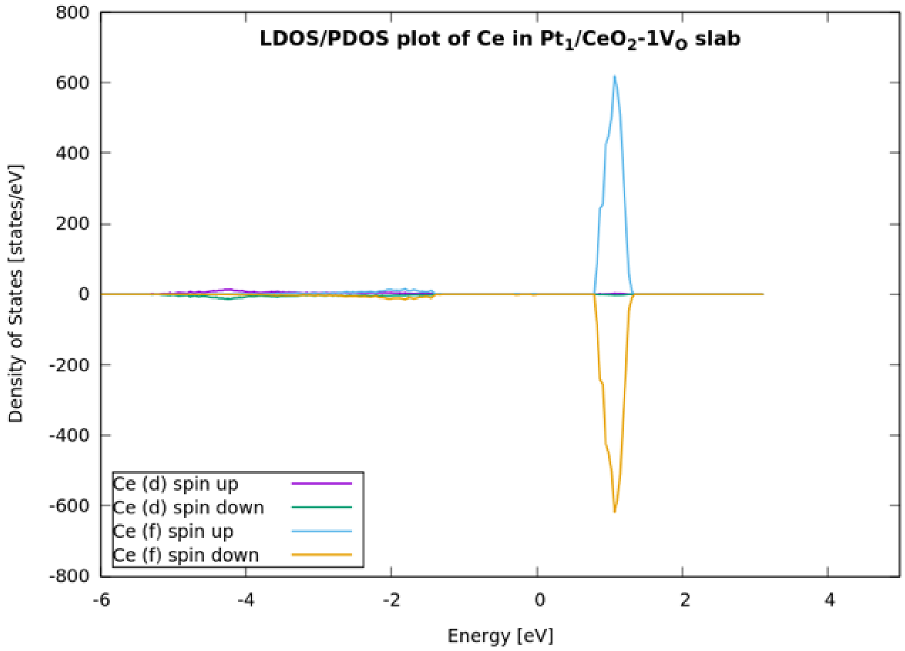

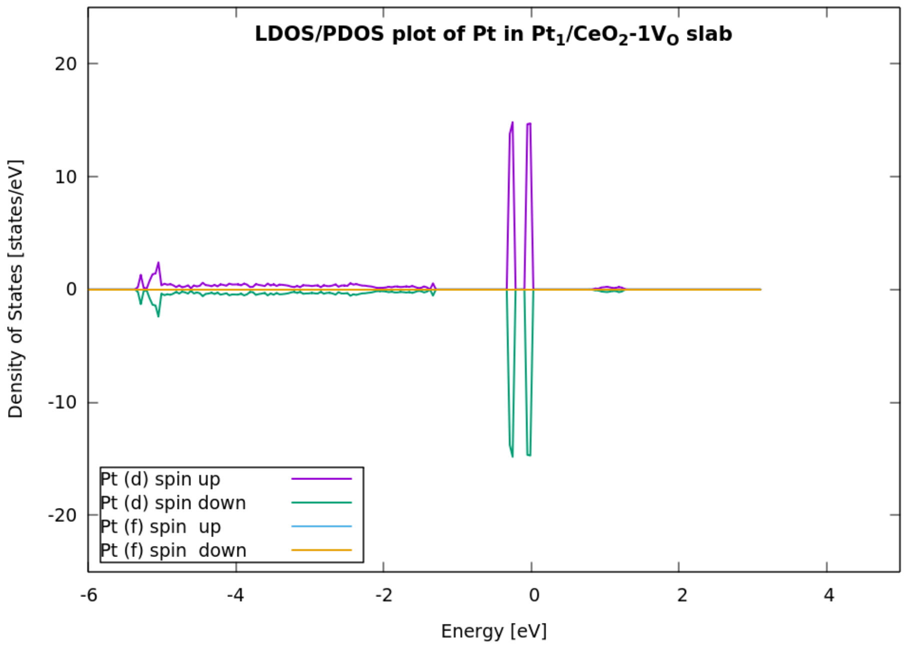

Supplementary Figure 20 Localized density of states (LDOS)/projected density of states (PDOS) plots of Ce and Pt ions in four systems, (a) CeO_2_slab, (b) CeO_2_$1V_O$ slab, (c) Pt_1/CeO_2_slab, and (d) Pt_1/CeO_2_$1V_O$ slab, displaying their d and f bands.

Supplementary References

(1) Mai, H. X. *et al.* Shape-selective synthesis and oxygen storage behavior of ceria nanopolyhedra, nanorods, and nanocubes. *J. Phys. Chem. B* **109**, 24380-24385 (2005).

(2) Rioux, R. M. *et al.* High-surface-area catalyst design: synthesis, characterization, and reaction studies of platinum nanoparticles in mesoporous SBA-15 silica. *J. Phys. Chem. B* **109**, 2192-2202 (2005).

(3) Zhang, Z. *et al.* Thermally stable single atom Pt/m-Al_2O_3 for selective hydrogenation and CO oxidation. *Nat. Comm.* **8**, 1-10 (2017).

(4) Ravel, B. *et al.* ATHENA, ARTEMIS, HEPHAESTUS: data analysis for X-ray absorption spectroscopy using IFEFFIT. *J. Synchrotron Radiat.* **12**, 537-541 (2005).

(5) Kresse, G. *et al.* Efficiency of Ab-Initio Total Energy Calculations for Metals and Semiconductors Using a Plane-Wave Basis Set. *Comput. Mater. Sci.* **6**, 15−50 (1996).

(6) Kresse, G. *et al.* Efficient Iterative Schemes for Abinitio Total-Energy Calculations Using a Plane-Wave Basis Set. *Phys. Rev. B: Condens. Matter Mater. Phys.* **54**, 11169−11186 (1996).

(7) Perdew, J. P. *et al.* Generalized Gradient Approximation Made Simple. *Phys. Rev. Lett.* **77**, 3865−3868 (1996).

(8) Grimme, S. *et al.* Effect of the Damping Function in Dispersion Corrected Density Functional Theory. *J. Comput. Chem.* **32**, 1456-1465 (2011).

(9) Liechtenstein, A. I. *et al.* Density-Functional Theory and Strong Interactions: Orbital Ordering in Mott-Hubbard Insulators. *Phys. Rev. B* **52**, R5467(R) (1995).

(10) Dudarev, S. L. *et al.* Electron-Energy-Loss Spectra and the Structural Stability of Nickel Oxide: An LSDA+U Study. *Phys. Rev. B* **57**, 1505 (1998).

(11) Dvořák, F. *et al.* Creating single-atom Pt-ceria catalysts by surface step decoration. *Nat Commun* **7,** 10801 (2016).

(12) Blöchl, P. E. Projector Augmented-Wave Method. *Phys. Rev. B: Condens. Matter Mater. Phys*. **50**, 17953−17978 (1994).

(13) Kresse, G. *et al.* From Ultrasoft Pseudopotentials to the Projector Augmented Wave Method. *Phys. Rev. B: Condens. Matter Mater. Phys*. **59**, 1758−1775 (1999).

(14) Tang, W. *et al.* A Grid-Based Bader Analysis Algorithm without Lattice Bias. *J. Phys.: Condens. Matter* **21**, 084204 (2009).

(15) Sanville, E. *et al.* An Improved Grid-Based Algorithm for Bader Charge Allocation. *J. Comp. Chem.* **28**, 899-908 (2007).

(16) Henkelman, G. *et al.* A Fast and Robust Algorithm for Bader Decomposition of Charge Density. *Comput. Mater. Sci.* **36**, 354-360 (2006).

(17) Yu, M. *et al.* Accurate and Efficient Algorithm for Bader Charge Integration. *J. Chem. Phys.* **134**, 064111 (2011).

(18) Biniwale, R. B. *et al.* Dehydrogenation of cyclohexane over Ni based catalysts supported on activated carbon using spray-pulsed reactor and enhancement in activity by addition of a small amount of Pt. *Catal. Lett.* **105**, 83−87 (2005).

(19) Pande, J. V. *et al.* Catalytic dehydrogenation of cyclohexane over Ag-M/ACC catalysts for hydrogen supply. *Int. J. Hydrogen Energy* **37**, 6756−6763 (2012).

(20) Nakaya, Y. *et al.* Active, Selective, and Durable Catalyst for Alkane Dehydrogenation Based on a Well-Designed Trimetallic Alloy. *ACS Catal.* **10**, 5163−5172 (2020).

(21) Mori, K. *et al.* Dehydrogenation of Methylcyclohexane over Zinc-Containing Platinum/Alumina Catalysts. *J. Jan. Petrol. Inst.* **61**, 350–356 (2018).
(22) Nakano, A. *et al.* Effects of Mn Addition on Dehydrogenation of Methylcyclohexane over Pt/Al_2O_3 Catalyst. *Appl. Catal. A Gen.* **543**, 75–81 (2017).
(23) Nagatake, S. *et al.* Dehydrogenation of Methylcyclohexane over Pt/TiO_2 Catalyst. *Catal. Lett.* **146**, 54–60 (2016).
(24) Yan, J. *et al.* Dehydrogenation of Methylcyclohexane over Pt-Sn Supported on Mg-Al Mixed Metal Oxides Derived from Layered Double Hydroxides. *Int. J. Hydrogen Energy* **43**, 9343–9352 (2018).
(25) Zhang, C. *et al.* Hydrogen Production by Catalytic Dehydrogenation of Methylcyclohexane over Pt Catalysts Supported on Pyrolytic Waste Tire Char. *Int. J. Hydrogen Energy* **36**, 8902–8907 (2011).
