# Supplementary material for: Reversible dehydrogenation and rehydrogenation of cyclohexane and methylcyclohexane by single-site platinum catalyst
Source: Nat Commun. 2022 Mar 1;13:1092. doi: 10.1038/s41467-022-28607-y (PMC8888751; doi:10.1038/s41467-022-28607-y)
Supplement: Supplementary file 2 — Peer Review File [file 41467_2022_28607_MOESM2_ESM.pdf]

Editorial Note: This manuscript has been previously reviewed at another journal that is not operating a transparent peer review scheme. The manuscript was considered suitable for publication without further review at *Nature Communications*.
